# Structural and Conformational Studies on Carboxamides of 5,6-Diaminouracils—Precursors of Biologically Active Xanthine Derivatives

**DOI:** 10.3390/molecules24112168

**Published:** 2019-06-09

**Authors:** Daniel Marx, Gregor Schnakenburg, Stefan Grimme, Christa E. Müller

**Affiliations:** 1 Pharmaceutical Institute, Pharmaceutical Chemistry I, University of Bonn, D-53121 Bonn, Germany; dmarx@uni-bonn.de; 2 Pharma Center Bonn, University of Bonn, D-53121 Bonn, Germany; 3 Department of Chemistry, Institute of Inorganic Chemistry, University of Bonn, D-53121 Bonn, Germany; gregor.schnakenburg@uni-bonn.de; 4 Mulliken Center for Theoretical Chemistry, Institute of Physical and Theoretical Chemistry, University of Bonn, D-53115 Bonn, Germany; grimme@thch.uni-bonn.de

**Keywords:** amide, DFT calculation, dynamic NMR, rotamers/conformers, uracil, xanthine, X-ray crystallography

## Abstract

8-Arylethynylxanthine derivatives are potent, selective adenosine A_2A_ receptor antagonists, which represent (potential) therapeutics for Parkinson’s disease, Alzheimer’s dementia, and the immunotherapy of cancer. 6-Amino-5-amidouracil derivatives are important precursors for the synthesis of such xanthines. We noticed an unexpected duplication of NMR signals in many of these uracil derivatives. Here, we present a detailed analytical study of structurally diverse 6-amino-5-carboxamidouracils employing dynamic and two-dimensional NMR spectroscopy, density functional theory calculations, and X-ray analysis to explain the unexpected properties of these valuable drug intermediates.

## 1. Introduction

**Figure 1:**
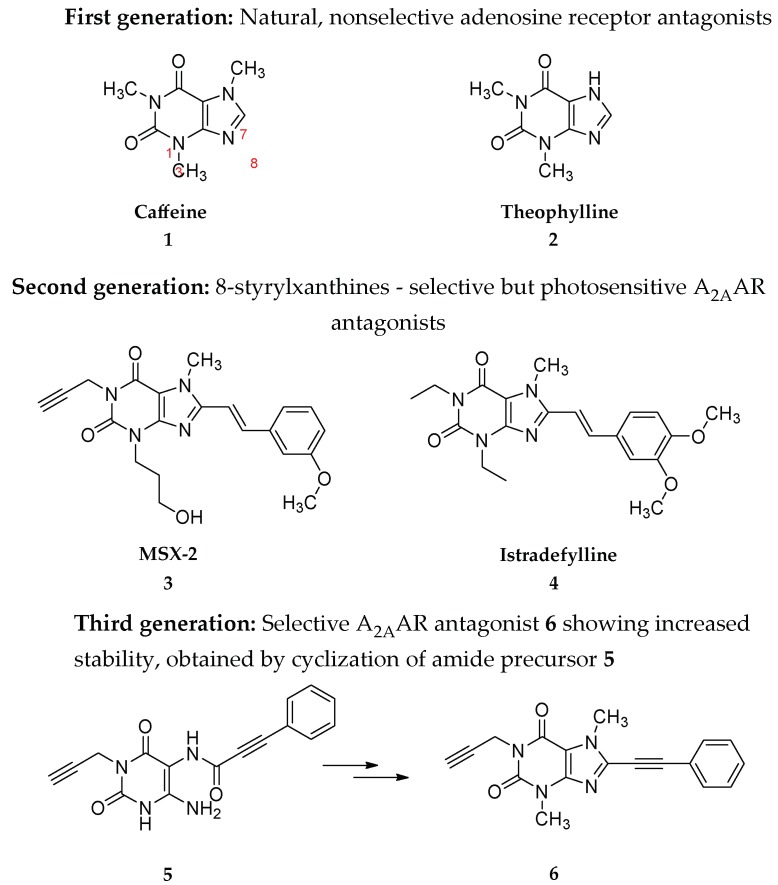
Examples of adenosine receptor antagonists with a xanthine scaffold. Shown is the development of the xanthine derivatives caffeine and theophylline towards potent and selective adenosine A_2A_ receptor antagonists.

The nucleoside adenosine is an important (patho)physiological modulator in the brain as well as in peripheral tissues and organs [1]. It activates G protein-coupled adenosine receptors (ARs) which comprise A_1_–, A_2A_–, A_2B_–, and A_3_AR subtypes. The A_2A_AR subtype has become a major (potential) drug target. A_2A_AR antagonists have been developed for neurodegenerative diseases such as Parkinson’s [2] and Alzheimer’s disease [3], and, recently, their great potential for the immunotherapy of cancer has been discovered [4]. The first identified AR antagonists were the natural alkaloids caffeine (1,3,7-trimethylxanthine, **1**) and theophylline (1,3-dimethylxanthine, **2**) (Figure 1) [5]. After discovering these alkylxanthines as moderately potent, non-selective AR antagonists, several groups tried to modify the xanthine scaffold to obtain more potent and selective compounds. *N*7-methylation, as in caffeine, combined with coplanar aromatic substitution at the *C*8-position of xanthines as in the styrylxanthines **3** and **4** (Figure 1) led to high affinity and selectivity for the A_2A_AR [6,7,8]. A disadvantage of the styrylxanthine structure is its photosensitivity in solid form as well as in dilute solutions [9].

**Scheme 1:**
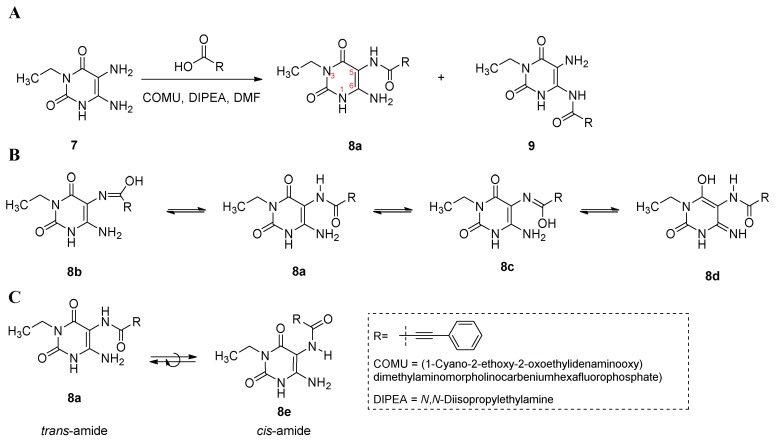
Potential products obtained by coupling of 5,6-diamino-3-ethyluracil **7** with phenylethynylcarboxylic acid. (**A**) Possible regioisomers **8a** and **9** that could result from an amide coupling reaction of **7** with phenylethynylcarboxylic acid. (**B**) Possible formation of tautomers **8b**, **8c**, and **8d** of **8a**. (**C**) Possible *cis*- and *trans*-amide bond conformers **8a** and **8e**.

**Figure 2:**
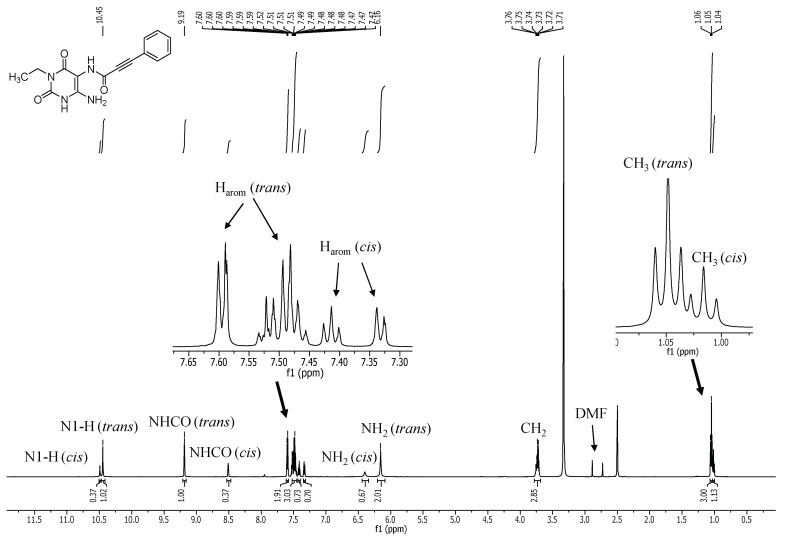
^1^H-NMR spectra of compound **8a** in DMSO-*d_6_*.

Replacement of the styryl moiety present in **3** and **4**, by an arylethynyl residue, as in **6**, resulted in a new class of photochemically stable A_2A_AR antagonists (Figure 1) [10]. Their 6-amino-5-carboxamidouracil precursors (e.g., compound **5**, Figure 1) were often not fully characterized, but directly converted to the final xanthine derivatives [11]. Upon close examination of ^1^H- and ^13^C-NMR spectra of 6-amino-5-arylethynylcarboxamidouracil **8a** (Scheme 1), obtained by coupling of 5,6-diamino-3-ethyl uracil **7** with phenylethynyl carboxylic acid, we observed a duplication of almost all signals (see Figure 2). However, this phenomenon was not observed for its styryl analog containing a double bond instead of the triple bond in **8a**. Thus, we performed a comprehensive analytical study of 6-amino-5-arylethynylcarboxamidouracil (**8a**) in order to rationalize these unexpected observations. Additionally, different *N*1,*N*3-substituted 6-amino-5-carboxamidouracil derivatives were synthesized and analyzed.

Different explanations for the duplication of peaks in the NMR spectra of 6-amino-5-arylethynylcarboxamides are conceivable. A mixture of regioisomers **8a** and **9** appeared to be likely due to possible amide coupling with the 5- or the 6-amino group of 5,6-diamino-3-ethyluracil (**7**, Scheme 1A). On the other hand, the presence of amide–iminol tautomers might be possible (Scheme 1B, structures **8a**, **8b**, **8c**, and **8d**). Moreover, amides can exist as different stereoisomers with s-*cis* (cisoid) or s-*trans* (transoid) conformation (see Scheme 1C), a phenomenon that is well known for peptides [12,13,14,15,16].

In the present study, we performed dynamic and 2D-NMR studies and X-ray crystallography, and additionally employed computational methods to elucidate the structures of 5/6-amino-5/6-carboxamidouracil derivatives such as **8**/**9** (Scheme 1). Different analogs of compound **8a** were synthesized and analyzed in detail in order to explore if different *N*1,*N*3-substituents or different linkers affect the structures leading to a duplication of NMR signals, as observed for compound **8**.

## 2. Results and Discussion

### 2.1. Chemistry

**Scheme 2:**
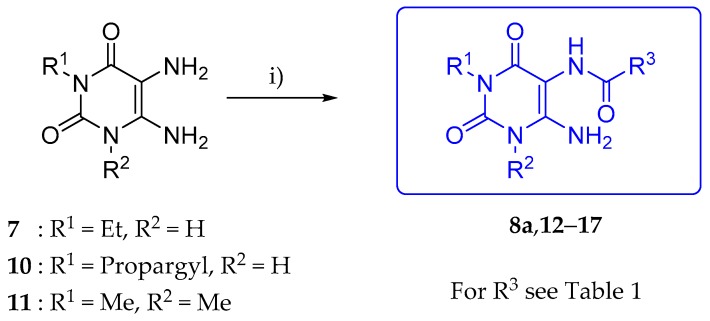
Synthesis of 6-amino-5-carboxamidouracil derivatives. Reagents and conditions: (i) R^3^-COOH, COMU, DIPEA, DMF, rt, 5 min, H_2_O (79%–90%). The synthesis of the starting material was performed according to procedures described in the literature (Scheme S1 and S2, Supplementary Information) [9].

Condensation of 5,6-diaminouracil derivatives **7**, **10**, and **11** with different carboxylic acid derivatives in the presence of (1-cyano-2-ethoxy-2-oxoethylidenaminooxy)dimethylamino-morpholinocarbenium hexafluorophosphate (COMU) yielded the desired 6-amino-5-carboxamido derivatives **8** and **12**–**17** (Scheme 2). The amide coupling reaction was performed by the recently described optimized procedure using COMU as a coupling reagent in the presence of diisopropylethylamine (DIPEA) as a base in dimethylformamide (DMF) at room temperature [17]. The products were precipitated by the addition of water, filtered off, and dried; no further purification was required. These precursors can be further converted to the corresponding xanthine derivatives by a dehydrative cyclization reaction [9,18,19].

### 2.2. Analytical Studies

In order to explain the duplication of signals in the NMR spectra of compound **8a** (Figure 2) different analytical experiments were performed. Yang et al. [20] described the formation of two regioisomers during the condensation reaction of 5,6-diamino-1,3-dimethyluracil with 2-hydroxyacetic acid. Furthermore, Poulsen et al. [21] proposed the formation of a 6-carboxamidouracil derivative during the condensation reaction of 5,6-diamino-1,3-dimethyluracil with glutamic anhydride. However, unambiguous evidence for the acylation of the 6-amino group was not provided by these authors.

High performance liquid chromatography (HPLC) of **8a** pointed to only a single product, a first indication that different regioisomers were likely not present, and the formation of **9** could be excluded (Scheme 1A). Additionally, compound **12** was selected for dynamic HPLC (DHPLC) experiments to explore the compound’s behavior at different temperatures. DHPLC measurements were performed at 5, 15, 25, and 40 °C, but in all cases one single peak was observed. Only a slight shift in the retention times and peak broadening was visible upon decreasing of the temperature (Figure S16, Supporting Information). To further elucidate whether regioisomers, tautomers, or conformational isomers were responsible for the signal duplication in the NMR spectra, dynamic NMR and 2D-NMR experiments were performed.

#### 2.2.1. Dynamic NMR Experiments of 8a at High Temperatures

**Figure 3:**
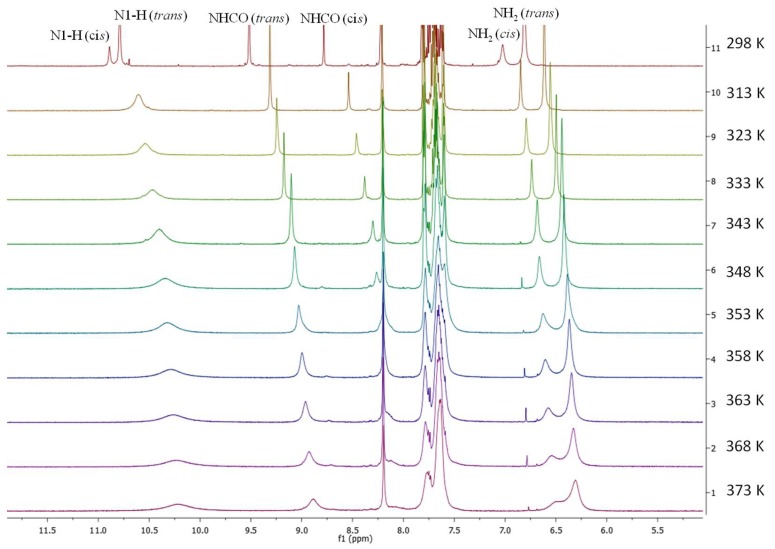
Dynamic NMR experiments of carboxamidouracil **8a** at high temperatures. The ^1^H-NMR spectra from 5.0 to 12.0 ppm of **8a** were measured in DMSO-*d*_6_ at different temperatures, between 298 and 373 K, to analyze the signals of the 6-amino group, the amide NH, and the N1-H in detail.

The most deshielded signal, that of the *N*1-H, resonates as a broad singlet at 10.43 ppm measured in DMSO-*d_6_*, and the second signal of the corresponding isomer is slightly shifted downfield to 10.48 ppm. The amide proton resonates at 9.17 ppm as a broad singlet, and the second signal shows a large upfield shift to 8.50 ppm. The amino group at position six resonates as a broad singlet at 6.14 ppm with an integration of two, while the corresponding second signal is shifted downfield to 6.39 ppm, showing an integration of 0.67. Dynamic NMR experiments were performed to study whether different tautomers of **8a** were present. The temperature should have a measurable influence on the ratio between tautomers. At a higher temperature, a higher percentage of the thermodynamically more stable tautomer would be expected [22]. ^1^H- and ^13^C-NMR spectra were recorded in DMF-*d*_7_ and DMSO-*d*_6_ from 223 to 378 K with 5–10 K intervals. The ratio of the isomers determined by integration of the ^1^H-NMR signals was found to be 73:27. Unexpectedly, in both solvents, no change in the ratio between the two species could be observed upon raising of the temperature (Figure 3). Since the dynamic NMR experiments showed that the existence of two tautomers next to each other in solution could be excluded (Scheme 1B), a third explanation was investigated in more detail. To confirm the third hypothesis of different conformers, 2D-NMR experiments in DMSO-*d*_6_ and DMF-*d*_7_ were performed.

#### 2.2.2. Two-Dimensional NMR Studies

**Figure 4:**
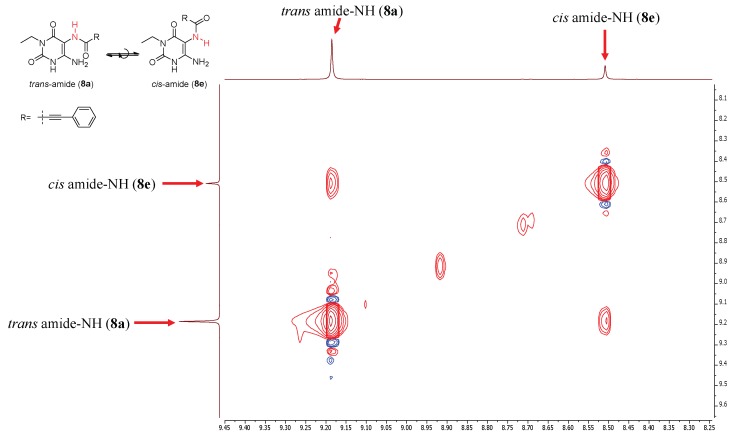
Exchange spectroscopy (EXSY)-NMR spectrum of **8a** in DMSO-*d*_6_ indicating cross-correlation in phase between the two amide-NH protons of conformer **8a** and **8e**. EXSY cross peaks of the *cis*-amide NH at 8.50 ppm and the *trans*-amide NH at 9.20 ppm are shown.

Nuclear Overhauser enhancement and exchange spectroscopy (NOESY and EXSY) experiments, showed the presence of cross peaks with the same phase as the diagonal peaks (experimental mixing time D8 of 300 ms, Figure 4) for almost all ^1^H signals, which points to a chemical exchange mechanism [12,23]. This takes place if a nucleus is transferred from one magnetic environment to another. As an example, the EXSY cross peaks of the *cis*-amide NH at 8.50 ppm and the *trans*-amide NH at 9.20 ppm are shown in Figure 4 in the same sign of the diagonal peaks (shown in red). Chemical exchange with the solvent could be excluded by the fact that we observed a cross-correlation also for non-exchangeable protons. Cross-correlation in phase of the signal of one conformer with the corresponding signal from the other conformer was observed as a proof for conformer equilibrium (Figure 4).

These results clearly showed that the duplication of peaks in the NMR spectra of **8a** (and related amidouracil derivatives) was due to the presence of different conformational isomers, such as **8a** and **8e** (Scheme 1C).

#### 2.2.3. Analysis of Differently Substituted 5-Amino-6-Carboxamidouracil Derivatives

We subsequently synthesized and investigated different 6-amino-5-carboxamidouracil derivatives with various carboxylic acid residues (Table 1) by dynamic and multidimensional NMR experiments. The equilibrium of their conformers and the energy of their rotational barrier were analyzed. In order to explore if the *N*1-substitution or the *N*3-substitution has any influence on the conformer equilibrium, compounds **12** (3-propargyl) and **13** (1,3-dimethyl) were synthesized (Table 1). Neither the change of the substitution pattern at the *N*1- or *N*3-position nor diethoxy substitution at the phenylethynyl residue changed the *cis*–*trans* ratio significantly (Table 1; compare **8a**, **12**, and **13**).

Finally, derivatives containing different linkers were prepared and analyzed. In the case of 8-arylethynyl derivatives the amount of the less stable conformer was between 20% and 30% in all three investigated cases (**8a**, **12**, and **13**). Mainly a single conformer was observed for the styryl-substituted derivative **14**, while with a single-bond linker in phenylethyl derivative **15** the ratio of *trans*- and *cis*-amide was approximately 89:11. A shorter linker shifted the equilibrium to a single conformer (**16**, **17**). However, this might also be due to a fast equilibrium between these two isomers, which cannot be distinguished by NMR spectroscopy. Due to the crystal structure and density functional theory (DFT) calculation of compound **8a** and the NOESY spectra of compound **17**, the *trans*-amide conformer is concluded to be the more stable rotational conformer.

#### 2.2.4. NMR Studies at Low Temperature

**Figure 5:**
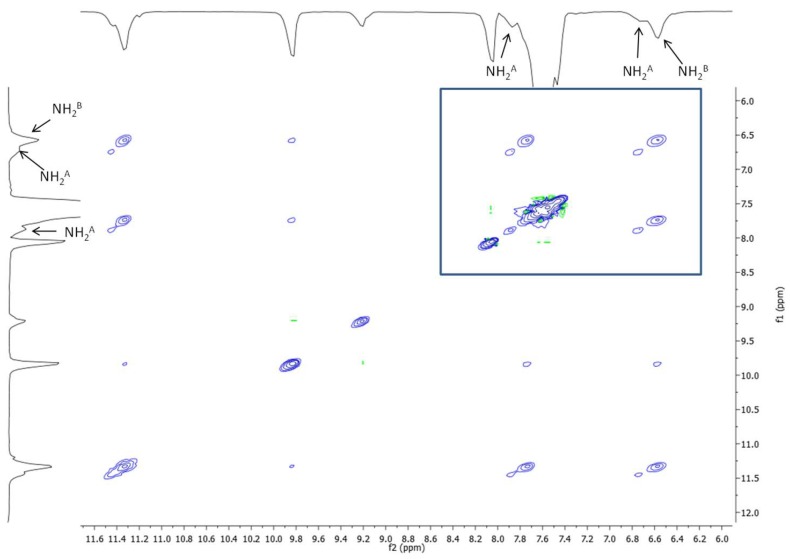
EXSY cross-correlation in DMSO-*d*_6_ suggesting a low rotational barrier of the C_sp_^2^–N bond in amide **8a** and **8e**. Cross-correlations for the amino groups of conformer A and conformer B were found at 6.76 and 7.89 ppm, and at 6.58 and 7.58 ppm, respectively.

**Figure 6:**
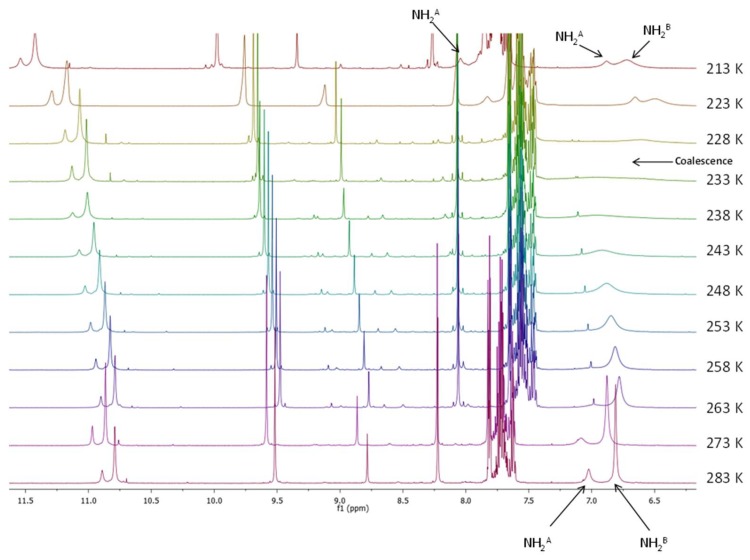
Proton dynamic NMR experiments of the carboxamidouracil isomers **8a** and **8e** in DMF-*d*_7_ at low temperature. Different NMR spectra for amide **8** were recorded from 213–283 K, with 5–10 K intervals. Coalescence of the 6-amino group was detected between 228 K and 233 K.

When further dynamic NMR experiments were performed for amide **8** at low temperature (213–283 K), a different conformer equilibrium was observed. NOESY spectra at 223 K showed the separation of the amino groups of both amide conformers **8a** and **8e** (Figure 5). A cross-correlation for the amino group of conformer A (compound **8a**) was found between two peaks at 6.76 and 7.89 ppm. The same cross peak was found for the amino group of conformer B (compound **8e**) at 6.58 and 7.58 ppm. The peak corresponding to the amino group of conformer B could only be observed with NOESY NMR because it was covered by the aromatic signals. The latter was expected to slow rotation of the C*sp*^2^–N bond [24]. The coalescence temperature of this amino group could be observed between 228 and 233 K (Figure 6). Using the simplified Eyring equation (Equation (4)), compound **8a** showed a rotational barrier for the amino group, ΔG^#^ = 46.4 ± 0.42 kJ·mol^−1^.

**Figure 7:**
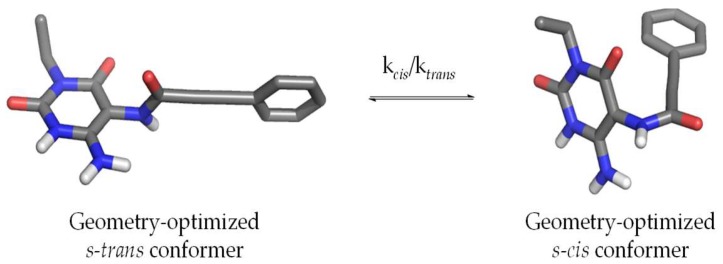
Geometry-optimized amide bond rotational conformers **8a** and **8e**. The conformers are shown in stick models (carbon atoms colored grey, oxygen atoms in red, the nitrogen atoms in blue, and the hydrogen atoms connected to nitrogen in white).

**Figure 8:**
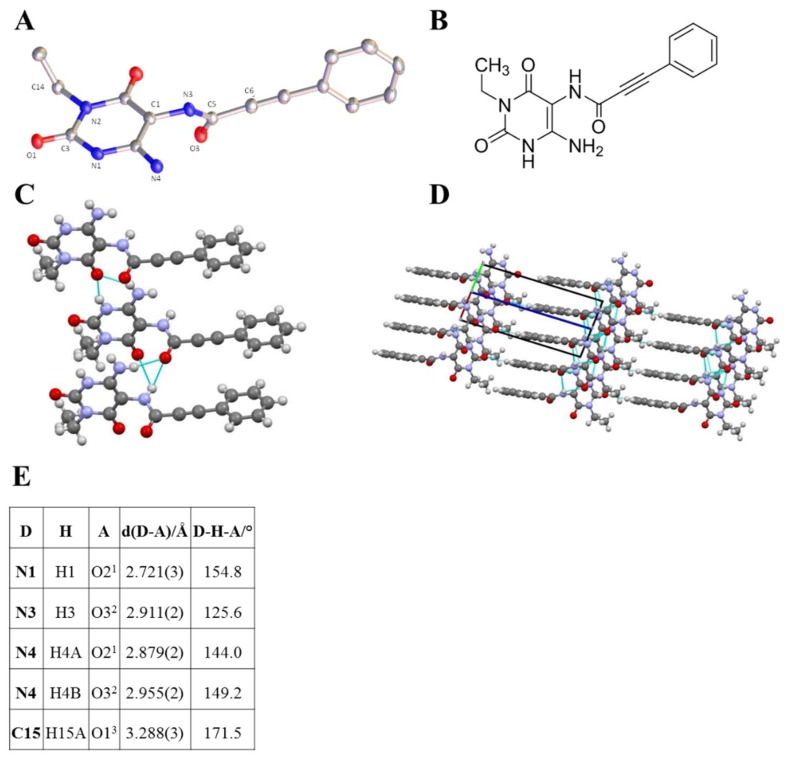
Results of the single X-ray crystallography of compound **8**. X-ray crystal structure (**A**) and chemical structure (**B**) of compound **8a**. (**C**) Intermolecular hydrogen bond interactions in the crystal (colored in cyan). (**D**) Crystal unit cell of the P1 space group. (**E**) Distances of intermolecular interactions in the crystal structure. For color coding see Figure 7.

However, the Eyring equation to calculate ΔG^#^ can only be used if the thermodynamic stability of the two conformers is equal. To determine ΔG^#^, quantum chemical calculations are required. A line-shape analysis was not possible due to the fact that the coalescence temperature was above 105 °C (see Figure 3). In order to predict the 3D structures of these conformers, density functional theory (DFT) calculations in liquid phase and X-ray crystallography for the solid state of **8a** were subsequently performed (Figure 7 and Figure 8).

#### 2.2.5. DFT Calculations

The DFT computed free energy difference between the two conformers is ΔG^#^ = −1 kcal, which corresponds to a k_trans_/k_cis_ ratio of 84:16 (Figure 7) at room temperature. This is consonant with the NMR results, which showed a ratio of 73:27 both indicating a higher stability of the *s*-*trans* conformer. The rotational barrier in DMSO solution is calculated to be ΔG# = 20.0 kcal·mol^−1^, which is in accordance with the results from the NMR experiments.

#### 2.2.6. X-ray Crystal Structure

Several solvents were tried to obtain single crystals, but finally we only obtained suitable crystals (triclinic space group P1) of compound **8a** from DMSO solution. The resulting crystal structure showed exclusively the more stable regioisomer **8a** (Figure 8). No intramolecular hydrogen bonding and no π-stacking between the molecules was observed. The crystals were found to be mainly formed by intermolecular hydrogen bonding as shown in Figure 8.

## 3. Conclusions

6-Amino-5-carboxamidouracil derivatives, which are important intermediates in the synthesis of pharmaceutically important 8-substituted xanthine derivatives, were observed to show a more or less pronounced duplication of NMR signals, depending on their carboxylic acid residue. In order to understand this phenomenon, selected 6-amino-5-carboxamidouracils were analyzed using dynamic and 2D NMR-experiments, DFT calculations, and single-molecule X-ray crystallography. The duplication of NMR signals could be correlated with a partial double bond character of the amide bond and a low rotational barrier of this bond depending on the carboxylic acid residue. According to DFT calculations, in the case of 5-ethynylcarboxamidouracils, the triple bond appears to stabilize the thermodynamically less stable *cis* conformer. This could be observed in solution, while the obtained crystal structure consisted solely of the more stable *trans* conformer.

## 4. Materials and Methods

Chemicals were purchased from Merck (Darmstadt, Germany), ABCR (Karlsruhe, Germany), or TCI (Eschborn, Germany). Thin layer chromatography (TLC) was performed on TLC plates F_254_ (Merck) and analyzed using UV light. High-resolution mass spectra (HR-MS) were recorded on a micrOTOF-Q mass spectrometer (Bruker, Billerica, MA, USA), further mass spectra were performed on an API 2000 (Applied Biosystems, Foster City, CA, USA) mass spectrometer. ^1^H- and ^13^C-NMR spectra were recorded in CDCl_3_, DMSO-*d_6_*, or DMF-*d*_7_ on a Bruker Ascend 600 MHz NMR-spectrometer (Bruker) operating at 600.18 MHz (^1^H) or 150.93 MHz (^13^C), respectively. Chemical shifts (δ) are reported in ppm and are referenced to the chemical shifts of the residual solvent proton(s) present in CHCl_3_ (7.26 ppm for the ^1^H-NMR spectra and 77.16 ppm for the ^13^C-NMR spectra), in DMSO (2.50 ppm for the ^1^H-NMR spectra and 39.52 ppm for the ^13^C-NMR spectra), and in DMF (2.75 ppm for the ^1^H-NMR spectra and 29.76 ppm for the ^13^C-NMR spectra). Multiplicity: s, singlet; d, doublet; q, quartet; m, multiplet. Coupling constants (*J*) are shown in Hertz (Hz). Dynamic HPLC analyses were performed on “Knauer GmbH” (Berlin, Germany) systems of the “PLATINblue” series with a P-1 pump, a column oven, a PDA-1 diode array UV-detector, and a Knauer Eurospher II 100-2 C18, (2 mm, 100 × 2.0 mm; number 19040303147) column. The solvents were of HPLC grade.

### 4.1. Eyring Equation

If rotation around an amide bond is slow on the NMR timescale, and the rotational barrier is between 3 and 19 kcal/mol, the observation of two conformers is permitted due to the partial double bond character of the amide C–N bond [25]. Under this assumption, one sharp peak for the duplicated signals of **8** should be observed at the coalescence temperature in the ^1^H-NMR spectrum [12,26,27,28]. Thus, the rotational barrier could be determined using the Eyring equations. Before the coalescence temperature is reached, the interconversion rate constant corresponds to the following equation:

(1) $$k=\frac{1}{t}\ll \pi \frac{∆\nu }{\sqrt{2}}$$

where k is interconversion rate constant (s^−1^), *t* is interconversion time (s), and ∆ν is the NMR shift (Hz) separation of the signals at low temperatures when exchange does not occur.

Heating leads to a faster exchange rate relative to the NMR timescale and only one averaged signal becomes detectable. At the coalescence temperature, the equation of the interconversion rate constant is

(2) $$k_{\mathrm{Tc}}=\pi \frac{∆\nu }{\sqrt{2}}$$

where k_Tc_ is the rate constant (s^−1^).

The free Gibbs activation energy ∆G# of rotation can be calculated using the following Eyring equations:

(3) $$-∆G^{\#}\;=\;Κ\frac{k_{B}\times T}{h}e^{\frac{-∆G^{\#}}{\mathrm{RT}}}$$

(4) $$∆G^{\#}=4.57{\times 10}^{-3}\;T\;(9.972+\log\frac{\mathrm{Tc}}{∆\nu })$$

where R is the universal gas constant ($\frac{1.0872\;\mathrm{cal}}{K\cdot \mathrm{mol}}$), *k_B_* is the Boltzmann constant (3.2998 × 10^−24^ $\frac{\mathrm{cal}}{K}$), h is the Planck constant (1.584 × 10^−34^ cal·s), and T_C_ is the coalescence temperature (K) [12].

### 4.2. DFT Calculation

Most of the quantum chemical calculations were carried out with the TURBOMOLE and ORCA programs [29,30]. All structures were fully optimized at the dispersion-corrected DFT level using the PBEh-3c DFT [31]. This composite method contains a modified Perdew–Burke–Ernzerhof (PBE)-based hybrid function together with an efficient valence double-zeta AO basis set. The method also involves an approximate counterpoise correction for basis set superposition errors (BSSE), as well as three-body dispersion effects [31]. Conformational searches [32], pre-optimizations, as well as the calculation of the Hessian to start transition state searches were conducted at the semi-empirical tight-binding level (GFN-xTB method) [33]. The GFN-xTB-optimized structures were used as input for subsequent full PBEh-3c optimizations. Reaction paths were obtained with the growing-string method (GSM) of Zimmerman [34] which was interfaced to our in-house XTB code [35]. Single-point gas phase energies were computed with the large polarized triple-zeta (def2-TZVPP) sets by Weigend et al. [36] in combination with the very accurate DSD-BLYP double hybrid functional [37]. The atom pairwise D3 correction with Becke–Johnson (BJ) damping to account for intra- and intermolecular London dispersion interactions was included in all treatments [38]. Note, that the original D2 treatment in DSD-BLYP is replaced by the D3 version (with damping parameters s6 = 0.57, a1 = 0, s8 = 0, a2 = 5.4) [39]. This functional performs excellently on the huge GMTKN55 thermochemical database (i.e., is practically the best out of 200+ tested DFT approximations) [40]. The combined level of theory used for electronic gas phase energies is denoted DSD-BLYP/TZ//PBEh-3c in standard notation. In all DFT treatments, the resolution-of-the-identity approximation [41] has been used for the two-electron integrals to speed up the computations. The numerical quadrature grid m4 (grid 5 in ORCA) was employed for the integration of the exchange-correlation contribution. Gibbs free energies at 298.15 K in DMSO were reported as a solvent (termed ΔG). The ro-vibrational corrections to the free energy are obtained from a modified rigid rotor, harmonic oscillator statistical treatment [42] based on scaled harmonic frequencies obtained at the (gas phase) HF-3c [43] level. For the entropy, all frequencies with wavenumbers below 100 cm^−1^ were treated as mixed rigid rotors and harmonic oscillators. Solvent effects on the thermochemical properties have been obtained by the COSMO-RS method [44] (COSMOtherm software package [44], parametrization from 2016) based on BP86/TZVP [36,45] single-point calculations. The PBEh-3c as well as GFN-xTB optimizations were run consistently in a continuum solvent. For GFN-xTB the built-in GBSA solvation model 23 is employed; while for PBEh-3c, the DCOSMO-RS method is used [46]. The solvation contributions to free energies at 298.15 K in DMSO solution are computed at those structures (i.e., PBEh-3c[DCOSMO-RS]) The computed free energies are obtained by ΔG = ΔE + ΔGRRHO + ΔδGCOSMO-RS, where the last two terms refer to the above mentioned ro-vibrational/translational and solvation contributions, respectively, to the free energy. The final theory level is denoted as DSD-BLYP/TZ[COSMO-RS(BP86/TZVP)]//PBEh-3c[DCOSMO-RS] where the abbreviations in square brackets denote the level of the solvation treatment.

### 4.3. General Procedures

#### 4.3.1. Synthesis of 5,6-Diaminouracil Derivatives

The different *N*-substituted 5,6-diaminouracils where synthesized in analogy to procedures described in the literature [9].

#### 4.3.2. General Procedure for Amide Formation

To a solution of the respective carboxylic acid (1.0 equiv.) and COMU (1.1 equiv.), dissolved in a minimum amount of DMF, a mixture of diaminouracil derivative (1.1 equiv.) and DIPEA (2.0 equiv.), dissolved in a minimum amount of DMF, was added dropwise. The reaction was stirred for 5–10 min at room temperature. Then, water was added, and the resulting precipitate was filtered off, washed with water, and dried under reduced pressure.

#### 4.3.3. NMR Spectra

*N*-(6-Amino-3-ethyl-2,4-dioxo-1,2,3,4-tetrahydropyrimidin-5-yl)-3-phenylpropiolamide (**8**). Yield: 85% (white solid); mp = 270–272 °C. Major isomer: ^1^H-NMR (600 MHz, DMSO-*d*_6_) δ 10.45 (s, 1H, N1-H), 9.16 (s, 1H, CONH), 7.62–7.56 (m, 2H, H_arom_), 7.53–7.45 (m, 3H, H_arom_), 6.14 (s, 2H, NH_2_), 3.79–3.67 (m, 2H, N1-CH_2_), 1.04 (t, *J* = 7.0 Hz, 2H, CH_2_CH_3_). ^13^C-NMR (126 MHz, DMSO-*d*_6_) δ 160.0 (CON), 152.7 (C6), 150.2 (CO), 149.5 (CO), 132.0 (2C, C_arom_), 130.2 (C_arom_), 129.0 (2C, C_arom_), 120.0 (C_arom_), 85.7 (C5 or C_alkyne_), 84.9 (C5 or C_alkyne_), 83.6 (C5 or C_alkyne_), 34.4 (N1-CH_2_), 13.2 (CH_2_CH_3_). Minor isomer: ^1^H-NMR (600 MHz, DMSO-*d*_6_) δ 10.49 (s, 1H, N1-H), 8.51 (s, 1H, CONH), 7.54–7.51 (m, 1H, H_arom_), 7.43–7.39 (m, 2H,H_arom_), 7.35–7.32 (m, 2H, H_arom_), 6.40 (s, 2H, NH_2_), 3.74–3.78 (m, 2H, N1-CH_2_), 1.01 (t, *J* = 7.0 Hz, 3H, CH_2_CH_3_). ^13^C-NMR (151 MHz, DMSO-*d*_6_) δ 162.3 (CON), 157.6 (C6), 151.5 (CO), 149.5 (CO), 132.0 (2C, C_arom_), 130.3 (C_arom_), 129.0 (2C, C_arom_), 120.0 (C_arom_), 87.5 (C5 or C_alkyne_), 85.8 (C5 or C_alkyne_), 83.2 (C5 or C_alkyne_), 34.4 (N1-CH_2_), 13.3 (CH_2_CH_3_). High resolution mass spectra (HRMS) (electrospray ionization-quadrupole-time-of-flight) (ESI-QTOF)) calculated for C_15_H_14_N_4_O_3_ [M + H]^+^: 299.1139; found: 299.1139.

*N*-(6-Amino-2,4-dioxo-3-(prop-2-yn-1-yl)-1,2,3,4-tetrahydropyrimidin-5-yl)-3-(3,4-diethoxyphenyl)propiolamide (**12**). Yield: 79% (white solid); mp = 185–183 °C. Major isomer: ^1^H-NMR (600 MHz, DMSO-*d*_6_) δ 10.64 (s, 1H, N1-H), 9.09 (s, 1H, CONH), 7.14 (dd, *J* = 8.4, 1.9 Hz, 1H, H_arom_), 7.09 (d, *J* = 2.1 Hz, 1H, H_arom_), 7.02 (d, *J* = 8.3 Hz, 1H, H_arom_), 6.25 (s, 2H, NH_2_), 4.41 (d, *J* = 2.4 Hz, 2H, H_propargyl_), 4.09–4.01 (m, 4H, 2 × OCH_2_), 3.02 (t, *J* = 2.4 Hz, 1H, H_propargyl_), 1.34–1.31 (m, 6H, 2 × OCH_2_CH_3_). ^13^C-NMR (151 MHz, DMSO-*d*_6_) δ 159.3, 153.0, 150.7, 150.2, 149.1, 147.9, 125.8 (C_arom_), 116.2 (C_arom_), 113.1 (C_arom_), 111.5 (C_arom_), 85.5 (C5 or C_alkyne_), 84.7 (C5 or C_alkyne_), 83.7 (C5 or C_alkyne_), 79.9 (C_propargyl_), 72.5 (C_propargyl_), 63.8 (2C, OCH_2_), 28.9 (N1-CH_2_), 14.6 (2C, OCH_2_CH_3_). Minor isomer: ^1^H-NMR (600 MHz, DMSO-*d*_6_) δ 10.72 (s, 1H, N1-H), 8.45 (s, 1H, CONH), 6.97–6.90 (m, 2H, H_arom_), 6.82 (d, *J* = 1.8 Hz, 1H, H_arom_), 6.50 (s, 2H, NH_2_), 4.51–4.44 (m, 2H, H_propargyl_), 4.05–4.02 (m, 2H, OCH_2_) 3.98 (q, *J* = 7.4 Hz, 2H, OCH_2_), 3.02–3.01 (m, 1H, H_propargyl_), 1.32–1.28 (m, 6H, OCH_2_CH_3_). ^13^C-NMR (151 MHz, DMSO-*d*_6_) δ 160.3, 157.8, 157.8, 151.9, 150.2, 149.1, 147.8, 126.0 (C_arom_), 116.2 (C_arom_), 113.0 (C_arom_), 111.4 (C_arom_), 87.4 (C5 or C_alkyne_), 87.1 (C5 or C_alkyne_), 81.8 (C5 or C_alkyne_), 79.9 (C_propargyl_), 72.4 (C_propargyl_), 63.9 (2C, OCH_2_), 28.9 (N1-CH_2_), 14.5 (2C, OCH_2_CH_3_). HRMS (ESI-QTOF) calculated for C_20_H_20_N_4_O_5_ [M + H]^+^: 397.1504; found: 397.1506.

*N*-(6-Amino-1,3-dimethyl-2,4-dioxo-1,2,3,4-tetrahydropyrimidin-5-yl)-3-(3,4-diethoxyphenyl)propiolamide (**13**). Yield: 88% (white solid); mp = 220–223 °C. Major isomer: ^1^H-NMR (600 MHz, DMSO-*d*_6_) δ 9.08 (s, 1H, CONH), 7.16 (dd, *J* = 8.3, 2.0 Hz, 1H, H_arom_), 7.11 (d, *J* = 1.9 Hz, 1H, H_arom_), 7.03 (d, *J* = 8.8 Hz, 2H, H_arom_), 6.76 (s, 2H, NH_2_), 4.07 (dq, *J* = 16.9, 7.0 Hz, 4H, 2 × OCH_2_), 3.31 (s, 3H, CH_3_), 3.12 (s, 3H, CH_3_), 1.34 (td, *J* = 7.0, 2.7 Hz, 6H, 2 × OCH_2_CH_3_). ^13^C-NMR (151 MHz, DMSO-*d*_6_) δ 158.9, 153.3, 152.1, 150.5, 150.2, 147.9, 125.8 (C_arom_), 116.2 (C_arom_), 113.1 (C_arom_), 111.6 (C_arom_), 86.1 (C5 or C_alkyne_), 84.5 (C5 or C_alkyne_), 83.9 (C5 or C_alkyne_), 63.9 (2C, OCH_2_), 30.0 (CH_3_), 27.5 (CH_3_), 14.6 (2C, OCH_2_CH_3_). Minor isomer: ^1^H-NMR (600 MHz, DMSO-*d*_6_) δ 8.46 (s, 1H, CONH), 7.04 (s, 1H, H_arom_), 6.96 (d, *J* = 8.4 Hz, 1H, H_arom_), 6.85 (dd, *J* = 8.3, 1.9 Hz, 1H, H_arom_), 6.75 (s, 2H, NH_2_), 4.07–4.05 (m, 2H, OCH_2_), 3.95 (q, *J* = 6.9 Hz, 2H, OCH_2_), 3.34 (s, 3H, CH_3_), 3.15 (s, 3H, CH_3_), 1.32–1.28 (m, 6H, 2 × OCH_2_CH_3_). ^13^C-NMR (151 MHz, DMSO-*d*_6_) δ 160.0, 158.0, 153.2, 150.5, 150.2, 147.9, 125.8 (C_arom_), 116.1 (C_arom_), 113.1 (C_arom_), 111.5 (C_arom_), 88.1 (C5 or C_alkyne_), 86.9 (C5 or C_alkyne_), 82.1 (C5 or C_alkyne_), 63.8 (2C, OCH_2_), 30.1 (CH_3_), 27.6 (CH_3_), 14.5 (2C, OCH_2_CH_3_). HRMS (ESI-QTOF) calculated for C_19_H_22_N_4_O_5_ [M + H]^+^: 387.1663; found: 387.1668.

*N*-(6-Amino-3-ethyl-2,4-dioxo-1,2,3,4-tetrahydropyrimidin-5-yl)cinnamamide (**14**). Yield: 80% (off-white solid); mp > 320 °C; ^1^H-NMR (500 MHz, DMSO-*d*_6_) δ 10.43 (s, 1H, N1-H), 8.68 (s, 1H, CONH), 7.58 (d, *J* = 7.4 Hz, 2H, H_arom_), 7.50–7.37 (m, 4H, H_arom_ + H_vinyl_), 6.83 (d, *J* = 15.9 Hz, 1H, H_vinyl_), 5.99 (s, 2H, NH_2_), 3.74 (q, *J* = 6.5 Hz, 2H, CH_2_), 1.06 (t, *J* = 6.7 Hz, 3H, CH_3_). ^13^C-NMR (DMSO-*d*_6_, 126 MHz) δ 164.9 (CON), 160.3 (C6), 149.7 (CO), 149.5 (CO), 138.8 (C_vinyl_ or C_arom_), 135.0 (C_vinyl_ or C_arom_), 129.4 (C_vinyl_ or C_arom_), 129.0 (2C, C_arom_), 127.4 (2C, C_arom_), 122.4 (C_vinyl_ or C_arom_), 87.4 (C5), 34.4 (N3-CH_2_), 13.2 (CH_3_). HRMS (ESI-QTOF) calculated for C_15_H_16_N_4_O_3_ [M + H]^+^: 301.1295; found: 301.1294.

*N*-(6-Amino-3-ethyl-2,4-dioxo-1,2,3,4-tetrahydropyrimidin-5-yl)-3-phenylpropanamide (**15**). Yield: 90% (white solid); mp > 320 °C; ^1^H-NMR (500 MHz, DMSO-*d*_6_) δ 10.38 (s, 1H, N1-H), 8.39 (s, 1H, CONH), 7.28 (t, *J* = 7.4 Hz, 2H, H_arom_), 7.24 (d, *J* = 6.9 Hz, 2H, H_arom_), 7.18 (t, *J* = 7.1 Hz, 1H, H_arom_), 5.82 (s, 2H, NH_2_), 3.73 (q, *J* = 6.9 Hz, 2H, N3-CH_2_), 2.91–2.80 (m, 2H, CH_2_), 2.53 (dd, *J* = 9.2, 7.0 Hz, 2H, CH_2_), 1.04 (t, *J* = 7.0 Hz, 3H, CH_3_). ^13^C-NMR (DMSO-*d*_6_, 126 MHz) δ 171.7 (CON), 160.4 (C6), 149.9 (CO), 149.6 (CO), 141.5 (C_arom_), 128.3 (2C, C_arom_), 128.1 (2C, C_arom_), 125.8 (C_arom_), 87.2 (C5), 36.8 (CH_2_), 34.3 (N3-CH_2_), 30.9 (CH_2_), 13.2 (CH_3_). HRMS (ESI-QTOF) calculated for C_15_H_18_N_4_O_3_ [M + H]^+^: 303.1452; found: 303.1454.

*N*-(6-Amino-3-ethyl-2,4-dioxo-1,2,3,4-tetrahydropyrimidin-5-yl)benzamide (**16**). Yield: 87% (yellowish solid; mp > 320 °C; ^1^H-NMR (500 MHz, DMSO-*d*_6_) δ 10.38 (s, 1H, N1-H), 8.86 (s, 1H, CONH), 7.99–7.91 (m, 2H, H_arom_), 7.56–7.51 (m, 1H, H_arom_), 7.47 (t, *J* = 7.5 Hz, 2H, H_arom_), 6.06 (s, 2H, NH_2_), 3.75 (q, *J* = 7.0 Hz, 2H, N3-CH_2_), 1.06 (t, *J* = 7.0 Hz, 3H, CH_3_). ^13^C-NMR (126 MHz, DMSO-*d*_6_) δ 166.4 (CON), 160.5 (C6), 150.4 (CO), 149.7 (CO), 134.5 (C_arom_), 131.1 (C_arom_), 128.0 (C_arom_), 127.8 (C_arom_), 87.1 (C5), 34.4 (N3-CH_2_), 13.3 (CH_3_). HRMS (ESI-QTOF) calculated for C_13_H_14_N_4_O_3_ [M + H]^+^: 275.1139; found: 275.1142.

*N*-(6-Amino-3-ethyl-2,4-dioxo-1,2,3,4-tetrahydropyrimidin-5-yl)-2-phenylacetamide (**17**). Yield: 80% (white solid); mp > 320 °C; ^1^H-NMR (500 MHz, DMSO-*d*_6_) δ 10.39 (s, 1H, N1-H), 8.58 (s, 1H, CONH), 7.35–7.31 (m, 2H, H_arom_), 7.28 (m, 2H, H_arom_), 7.23–7.19 (m, 1H, H_arom_), 5.90 (s, 2H, NH_2_ ), 3.71 (q, *J* = 7.0 Hz, 2H, N3-CH_2_), 3.56 (s, 2H, CH_2_), 1.03 (t, *J* = 7.0 Hz, 3H, CH_3_). ^13^C-NMR (DMSO-*d*_6_, 126 MHz) δ 170.6 (CON), 160.5 (C6), 150.1 (CO), 149.7 (CO), 136.6 (C_arom_), 129.4 (C_arom_), 128.2 (C_arom_), 126.3 (C_arom_), 87.5 (C5), 42.1 (COCH_2_), 34.5 (N3-CH_2_), 13.4 (CH_3_). HRMS (ESI-QTOF) calculated for C_14_H_16_N_4_O_3_ [M + H]^+^: 289.1295; found: 289.1304.

## Figures and Tables

**Table 1:** Synthesized 6-amino-5-carboxamidouracil derivatives, yields, and experimental *trans*/*cis* ratios from ^1^H-NMR in DMSO-*d*_6_.

Compound	Structure	Yield ^a^	*trans*/*cis* % Ratio ^b^
**8a**	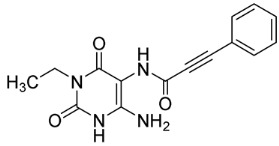	85%	73:27
**12**	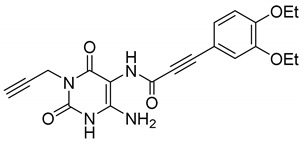	79%	72:28
**13**	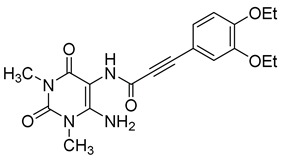	88%	77:23
**14**	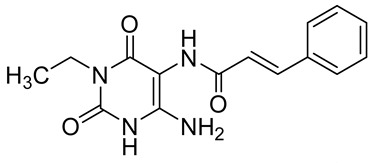	80%	95:5
**15**	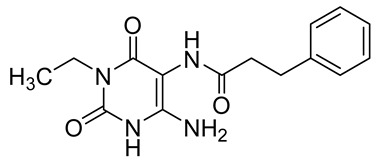	90%	89:11
**16**	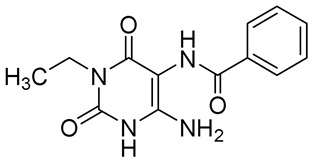	87%	100:0
**17**	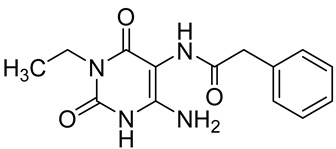	80%	91:9

^a^ Isolated yields of the amide coupling reaction of 5,6-diaminouracil derivatives (**7**, **10**, and **11**) with different carboxylic acid derivatives. ^b^ The ratio of the *cis*- and *trans*-amide conformers of **8a** and **12**–**17** were determined by integration of their ^1^H-NMR signals.

## Supplementary Materials

The following data are available online. Figure S1–S15: NMR spectra, Figure S16: DHPLC analyses of **12** at 205 nm, Figure S17: UV-spectrum of compound **12**. Table S1–S3: Crystal data and structure refinement for compound **8**. Scheme S1–S2: Synthesis of diaminouracil derivatives.
