# Supplementary material for: Structural and Conformational Studies on Carboxamides of 5,6-Diaminouracils—Precursors of Biologically Active Xanthine Derivatives
Source: Molecules. 2019 Jun 9;24(11):2168. doi: 10.3390/molecules24112168 (PMC6600361; doi:10.3390/molecules24112168)
Supplement: Supplementary file 1 [file molecules-24-02168-s001.pdf]

Supplementary Materials

Structural and conformational studies on carboxamides of 5,6-diaminouracils – precursors of biologically active xanthine derivatives

Daniel Marx,[1,2] Gregor Schnakenburg,[3] Stefan Grimme,[4] Christa E. Müller*[1,2]

*Correspondence: Prof. Dr. Christa E. Müller, christa.mueller@uni-bonn.de, +49 (0) 228 / 73-2301

TABLE OF CONTENTS

Synthesis of diaminouracil derivatives 2

Crystallographic data 3

^{1}H- and ^{13}C-NMR spectra of compound **8, 12–17**.. 8

Synthesis of diaminouracil derivatives

*N*1- and *N*3-substituted 5,6-diaminouracils were used as starting materials for the preparation of the corresponding 6-amino-5-carboxamidouracil derivatives and synthesized according to literature procedures [1-4].

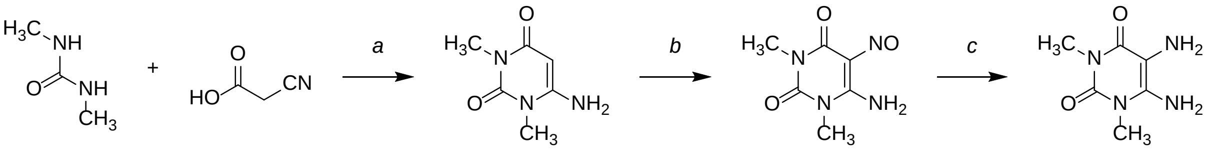

11

Scheme S1. Synthesis of 5,6-diamino-1,3-dimethyluracil. Reagents and conditions: (a) Ac_2O, 60 °C, 3 h; (b) aq. AcOH, HNO_2, 50-60 °C; (c) sodium dithionite, NH_3/H_2O, 60 °C.

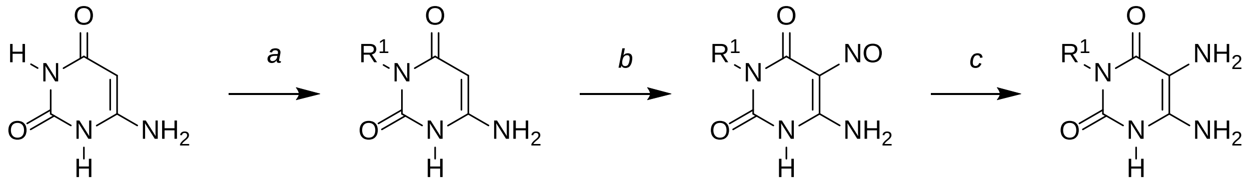

7 and **10**

7 R_1 = ethyl
10 R^1 = propargyl

Scheme S2. Synthesis of *N*3-substituted 5,6-diaminouracil derivatives **7** and **10**. Reagents and conditions: (a) 2.1 equiv of hexamethyldisilazane (HMDS), reflux, 60–70 °C, 1.7 equiv of ethyl iodide for (**7**) or 3-bromopropyne for (**10**); (b) aq AcOH, HNO_2, 50-60 °C; (c) sodium dithionite, NH_3/H_2O, 60 °C.

Analytical data were in accordance with published data. For details see [1, 2, 4].

Crystallographic data

Table S1. Crystal data and structure refinement for compound 8.

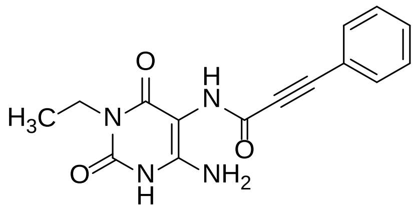

8

Identification code	GPHARM63, **8** // GXray5352
Crystal Habitus	clear colourless block
Device Type	STOE IPDS-2T
Empirical formula	$C_{15}H_{14}N_4O_3$
Moiety formula	C15 H14 N4 O3
Formula weight	298.30
Temperature/K	123(2)
Crystal system	triclinic
Space group	P1
a/Å	4.6523(3)
b/Å	5.5387(4)
c/Å	13.7337(9)
α/°	82.818(5)
β/°	89.265(5)
γ/°	80.921(6)
Volume/Å^3	346.70(4)
Z	1
ρ_{calc}g/cm^3	1.429

μ/mm^{-1}	0.103
F(000)	156.0
Crystal size/mm^3	0.32 × 0.3 × 0.24
Absorption correction	none
Radiation	MoKα (λ = 0.71073)
2Θ range for data collection/°	5.98 to 50.498°
Completeness to theta	0.999
Index ranges	-5 ≤ h ≤ 5, -6 ≤ k ≤ 6, -16 ≤ l ≤ 16
Reflections collected	8705
Independent reflections	2406 [R_{int} = 0.0736, R_{sigma} = 0.0473]p
Data/restraints/parameters	2406/3/201
Goodness-of-fit on F^2	1.070
Final R indexes [I>=2σ (I)]	R_1 = 0.0281, wR_2 = 0.0730
Final R indexes [all data]	R_1 = 0.0297, wR_2 = 0.0735
Largest diff. peak/hole / e $Å^{-3}$	0.16/-0.15
Flack parameter	0.5(6)

Table S2 Bond Lengths for compound 8.

Atom	Atom	Length/Å	Atom	Atom	Length/Å
O1	C3	1.214(3)	C1	C4	1.415(3)
O2	C4	1.249(3)	C5	C6	1.449(3)
O3	C5	1.231(3)	C6	C7	1.201(3)
N1	C2	1.367(3)	C7	C8	1.436(3)
N1	C3	1.375(3)	C8	C9	1.392(3)
N2	C3	1.384(3)	C8	C13	1.394(3)
N2	C4	1.401(3)	C9	C10	1.387(3)
N2	C14	1.475(3)	C10	C11	1.373(4)
N3	C1	1.419(3)	C11	C12	1.381(4)

N3	C5	1.347(3)	C12	C13	1.388(3)
N4	C2	1.339(3)	C14	C15	1.518(3)
C1	C2	1.375(3)			

Table S3 Bond Angles for compound 8.

Atom	Atom	Atom	Angle/°	Atom	Atom	Atom	Angle/°
C2	N1	C3	124.8(2)	N2	C4	C1	117.38(19)
C3	N2	C4	122.84(17)	O3	C5	N3	123.72(19)
C3	N2	C14	116.45(18)	O3	C5	C6	122.2(2)
C4	N2	C14	120.6(2)	N3	C5	C6	114.1(2)
C5	N3	C1	122.34(19)	C7	C6	C5	177.1(2)
C2	C1	N3	120.22(18)	C6	C7	C8	179.0(2)
C2	C1	C4	120.75(18)	C9	C8	C7	119.9(2)
C4	C1	N3	119.03(19)	C9	C8	C13	120.1(2)
N1	C2	C1	118.26(18)	C13	C8	C7	120.0(2)
N4	C2	N1	116.4(2)	C10	C9	C8	119.6(2)
N4	C2	C1	125.34(19)	C11	C10	C9	120.1(2)
O1	C3	N1	120.9(2)	C10	C11	C12	120.7(2)
O1	C3	N2	123.25(18)	C11	C12	C13	120.1(2)
N1	C3	N2	115.87(18)	C12	C13	C8	119.4(2)
O2	C4	N2	119.22(19)	N2	C14	C15	112.32(19)
O2	C4	C1	123.40(19)				

^{1}H- and ^{13}C-NMR spectra of compounds **8, 12-17**.

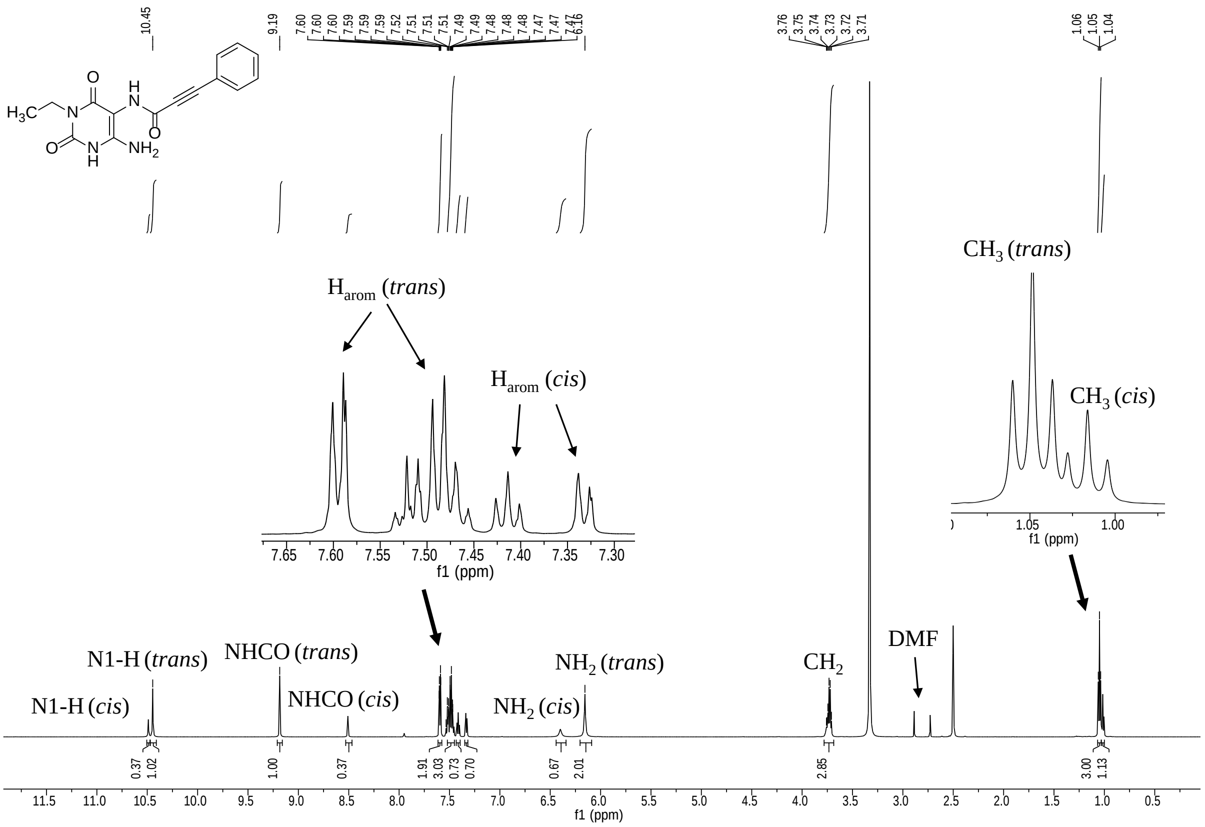

Figure S1. ^{1}H-NMR spectra of **8** in $(CD_3)_2SO$ at room temperature.

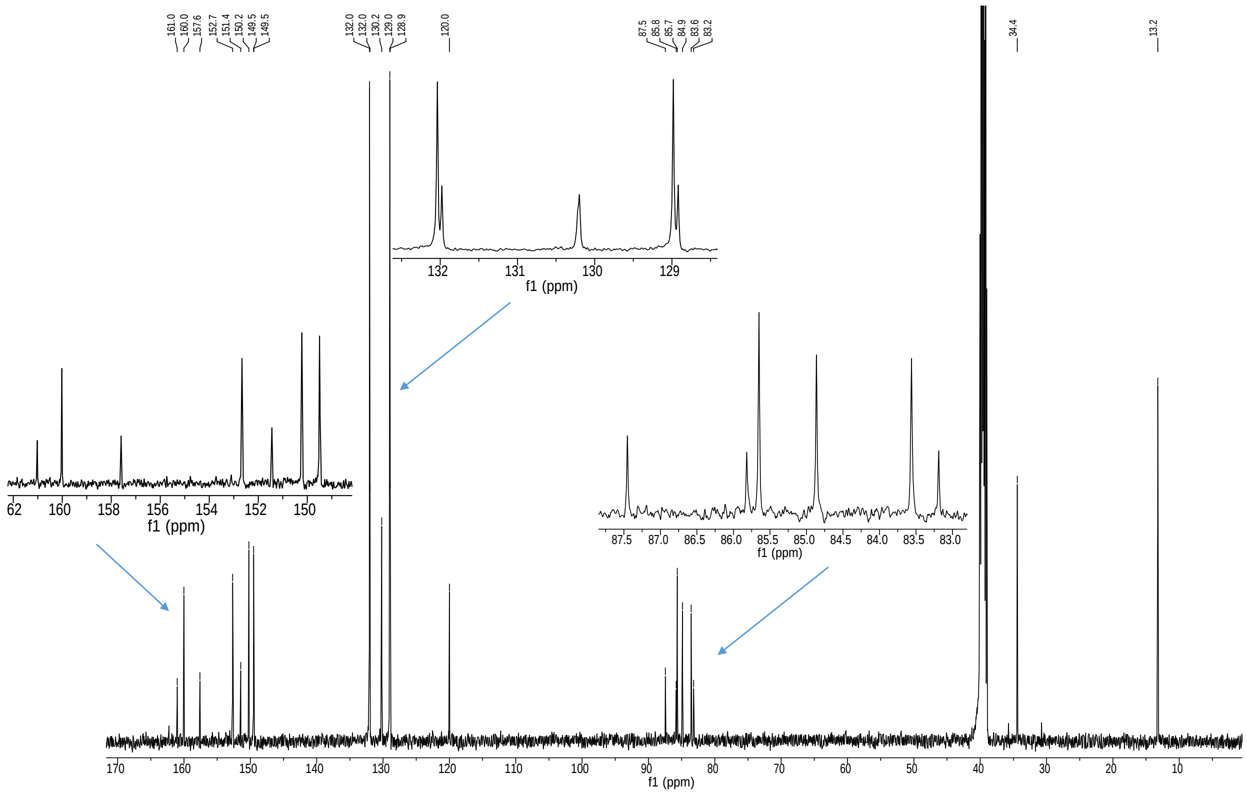

Figure S2. ^{13}C-NMR spectra of **8** in $(CD_3)_2SO$ at room temperature.

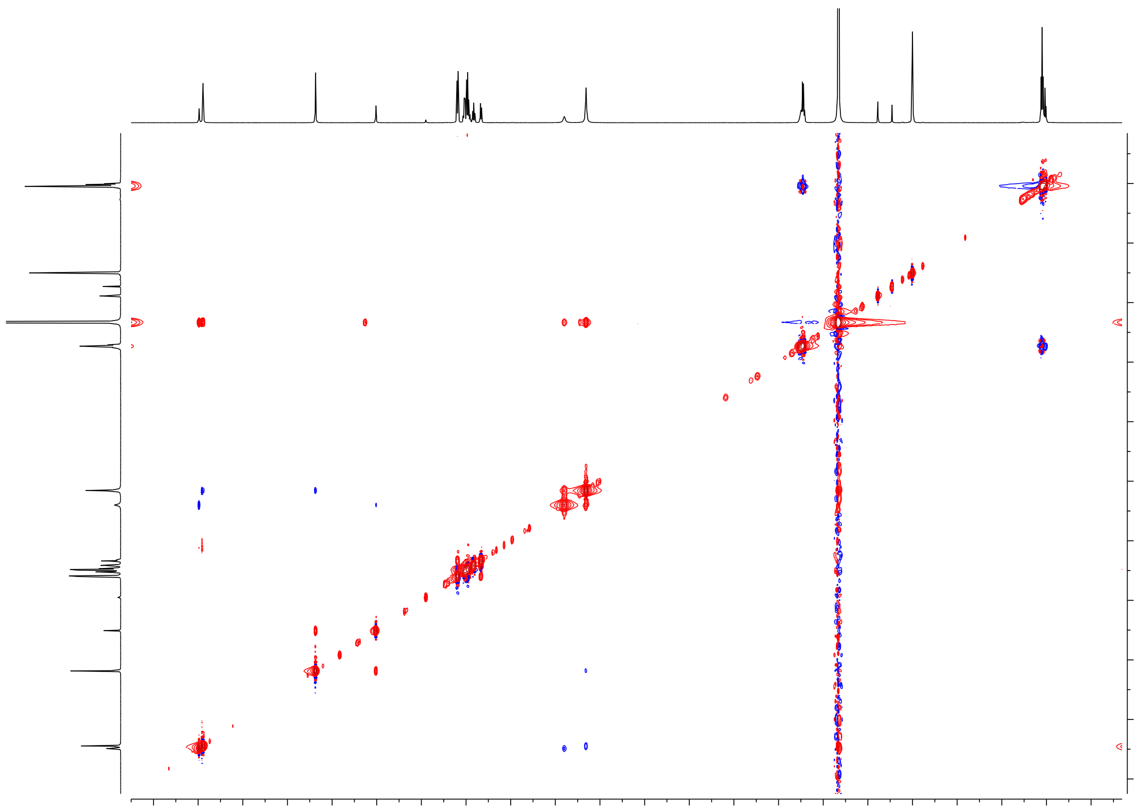

Figure S3. NOESY-NMR spectra of **8** in $(CD_3)_2SO$ at room temperature.

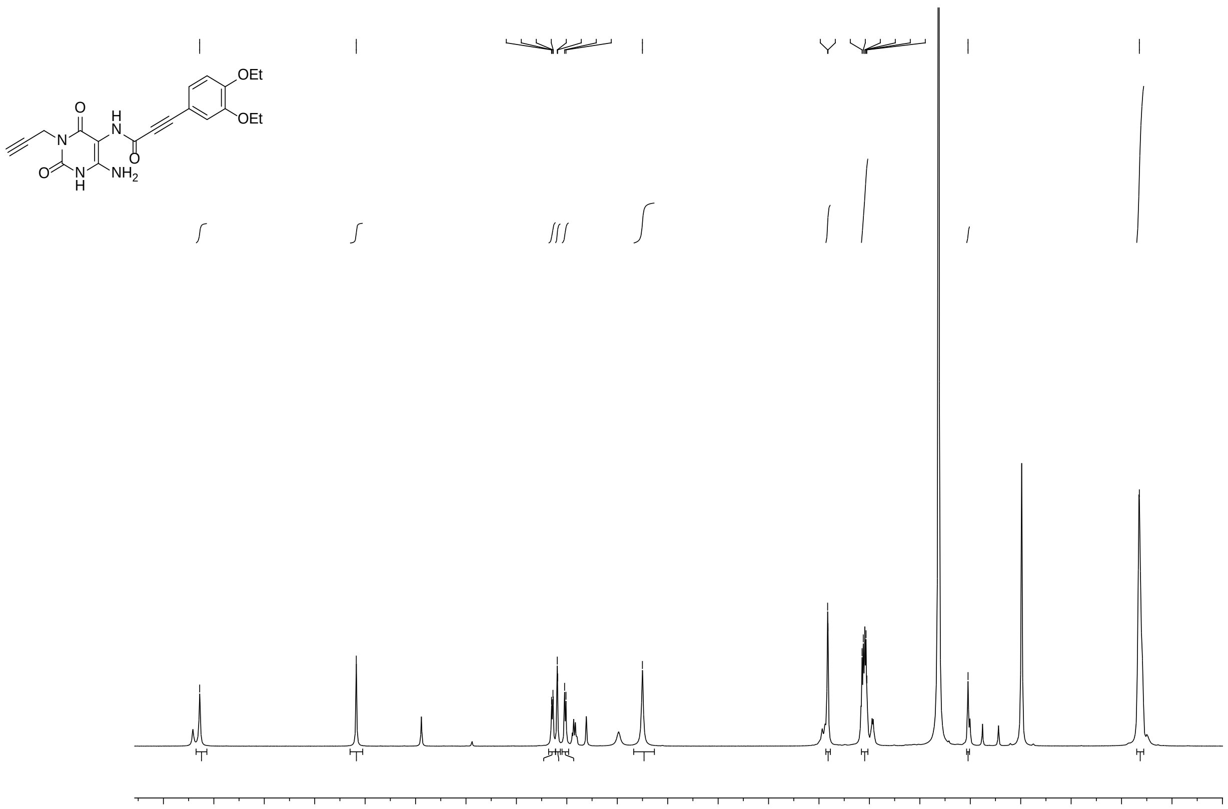

Figure S4. ^1H-NMR spectra of **12** in $(CD_3)_2SO$ at room temperature.

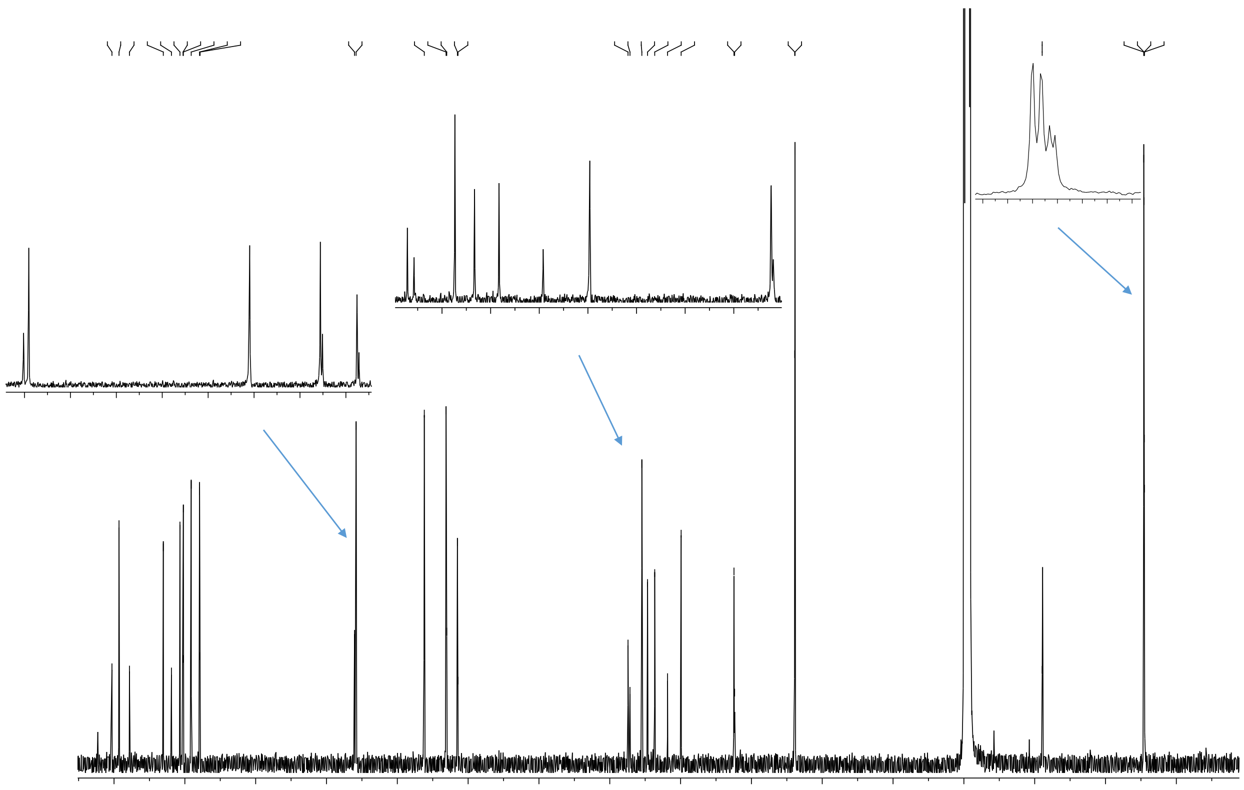

Figure S5. ^{13}C-NMR spectra of **12** in $(CD_3)_2SO$ at room temperature.

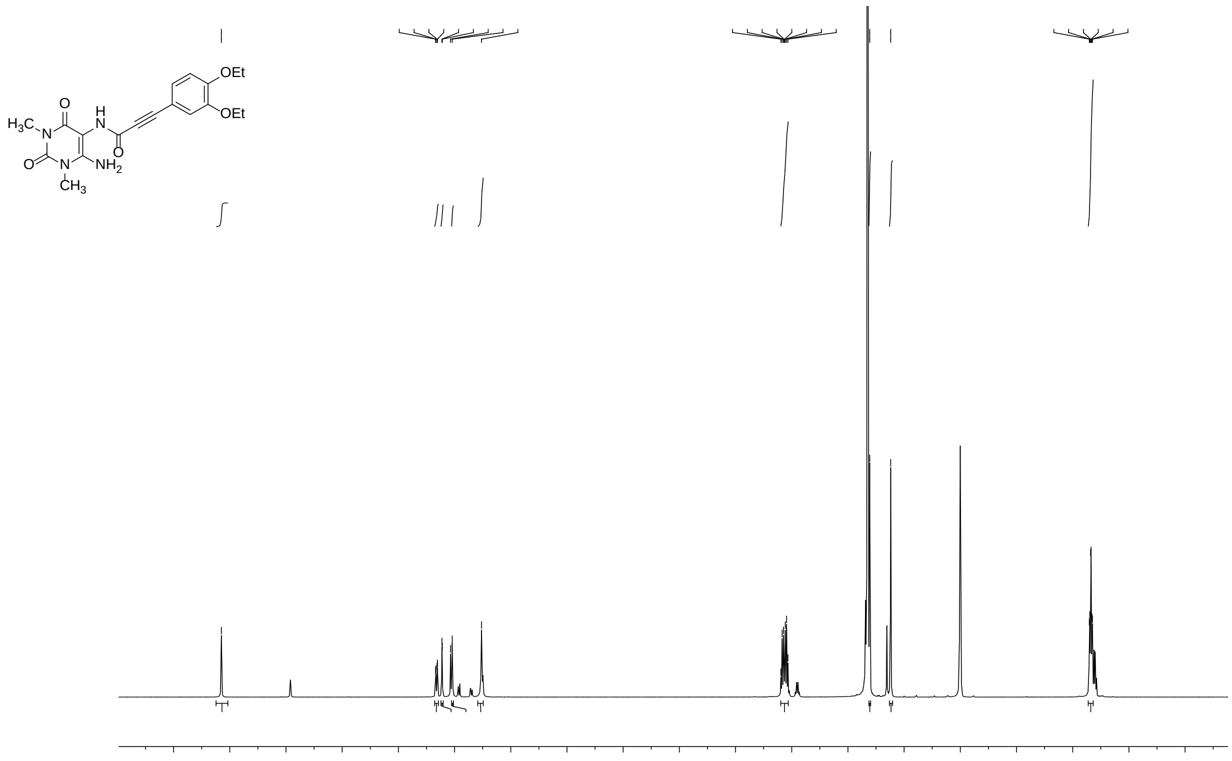

Figure S6. ^{1}H-NMR spectra of **13** in $(CD_3)_2SO$ at room temperature.

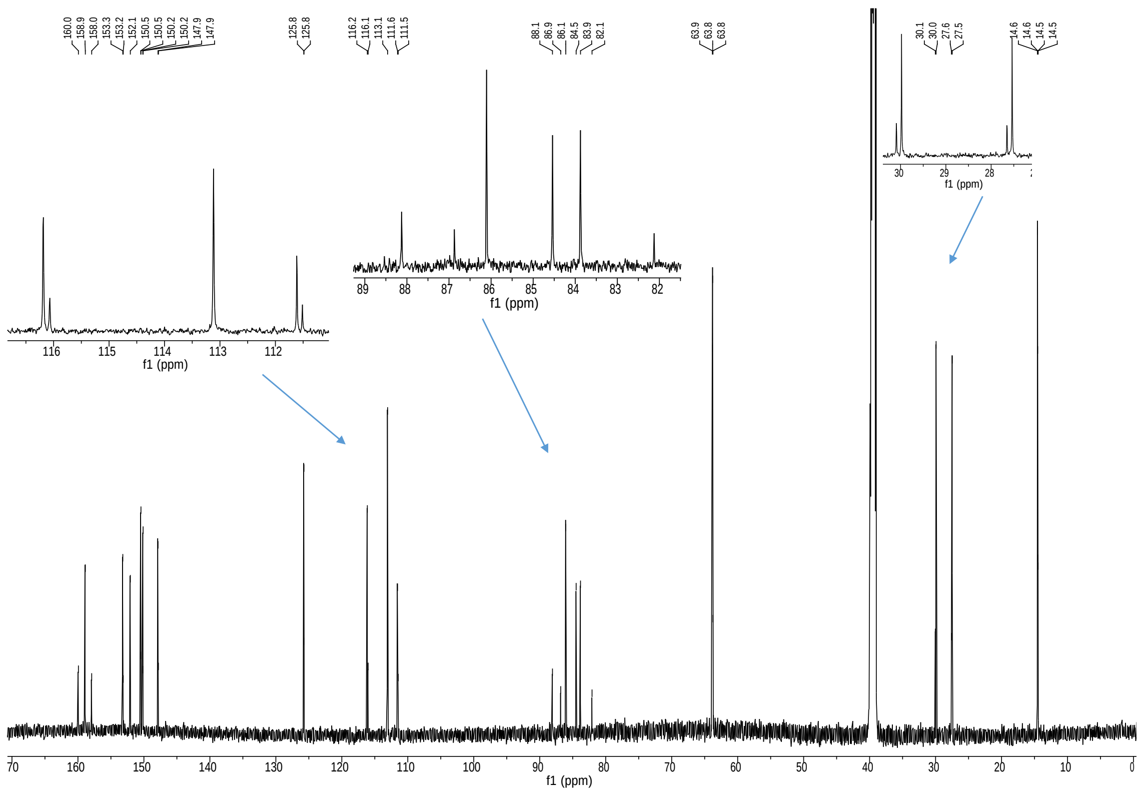

Figure S7. ^{13}C-NMR spectra of **13** in $(CD_3)_2SO$ at room temperature.

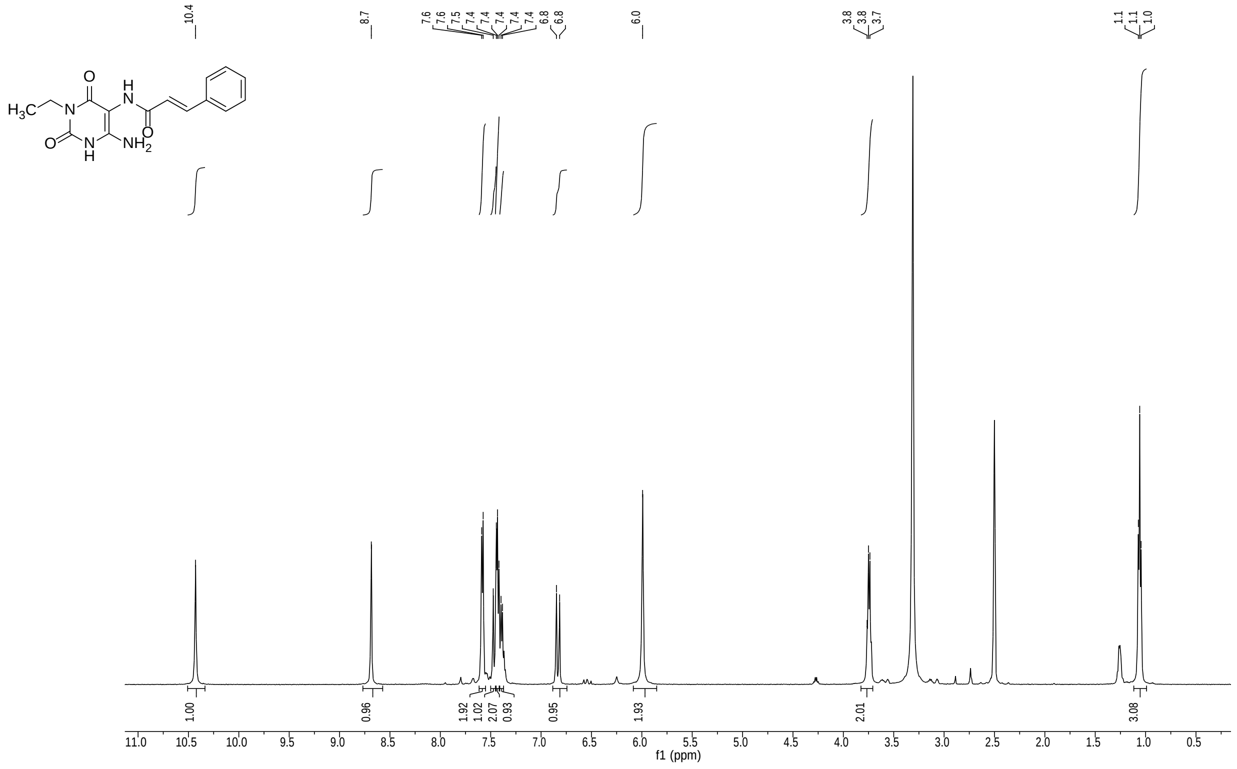

Figure S8. ^{1}H-NMR spectra of **14** in $(CD_3)_2SO$ at room temperature.

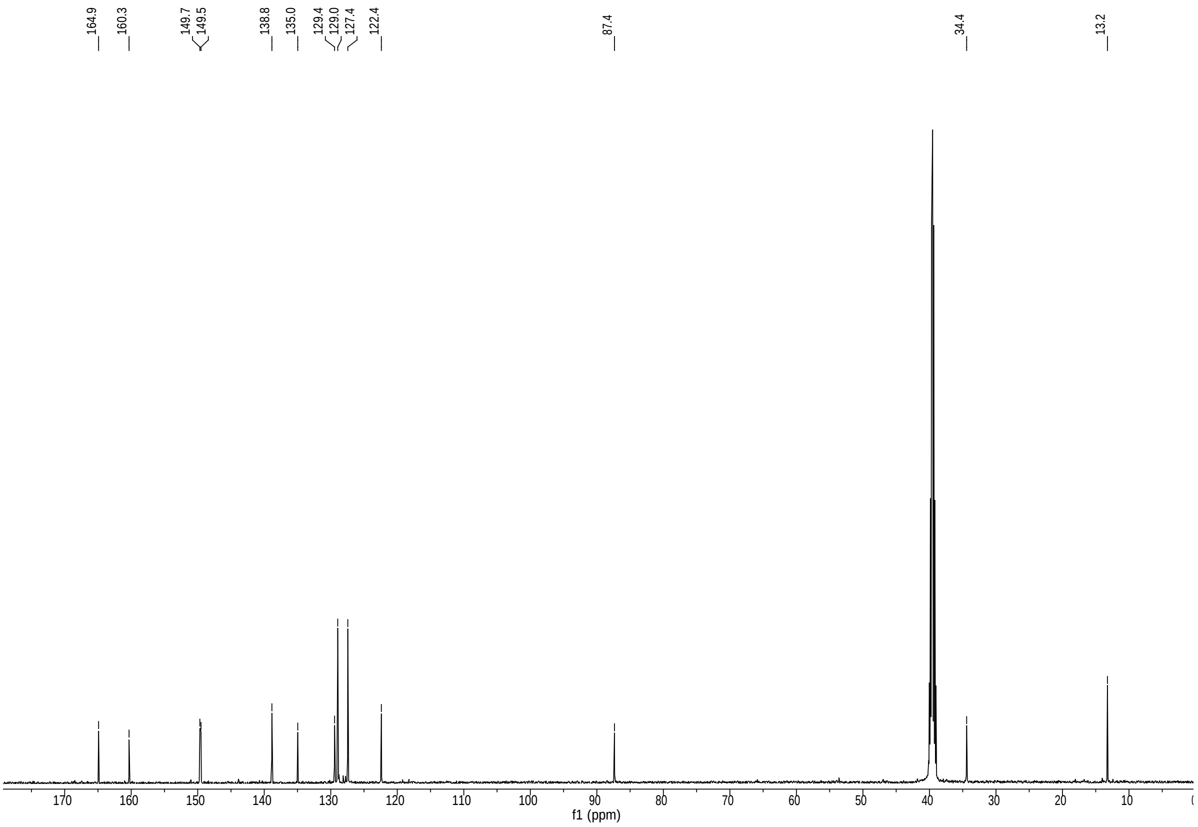

Figure S9. ^{13}C-NMR spectra of **14** in $(CD_3)_2SO$ at room temperature.

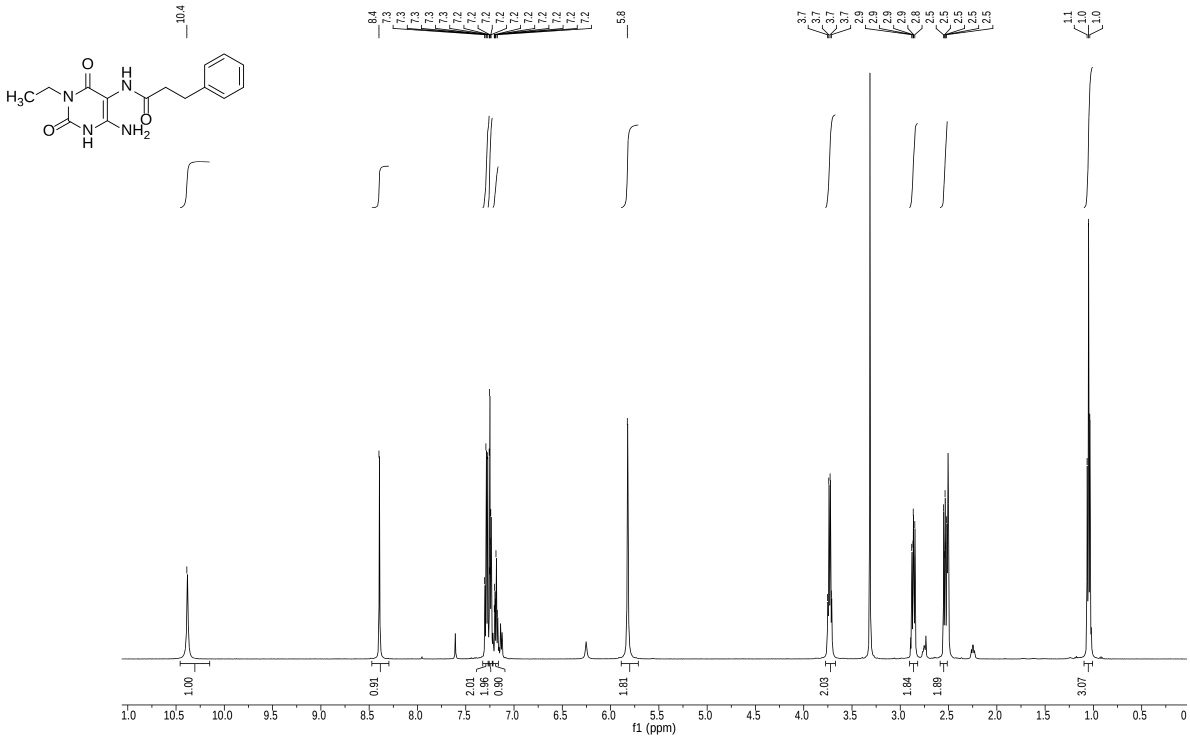

Figure S10. ^{1}H-NMR spectra of **15** in $(CD_3)_2SO$ at room temperature.

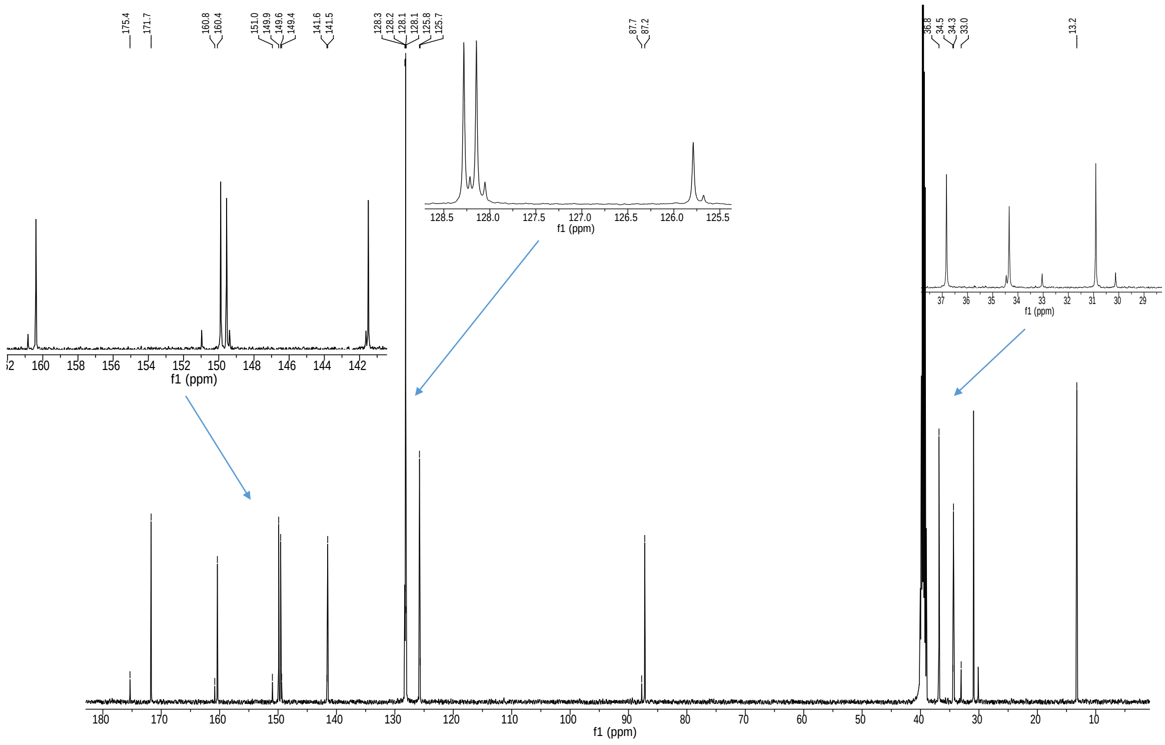

Figure S11. ^{13}C-NMR spectra of **15** in $(CD_3)_2SO$ at room temperature.

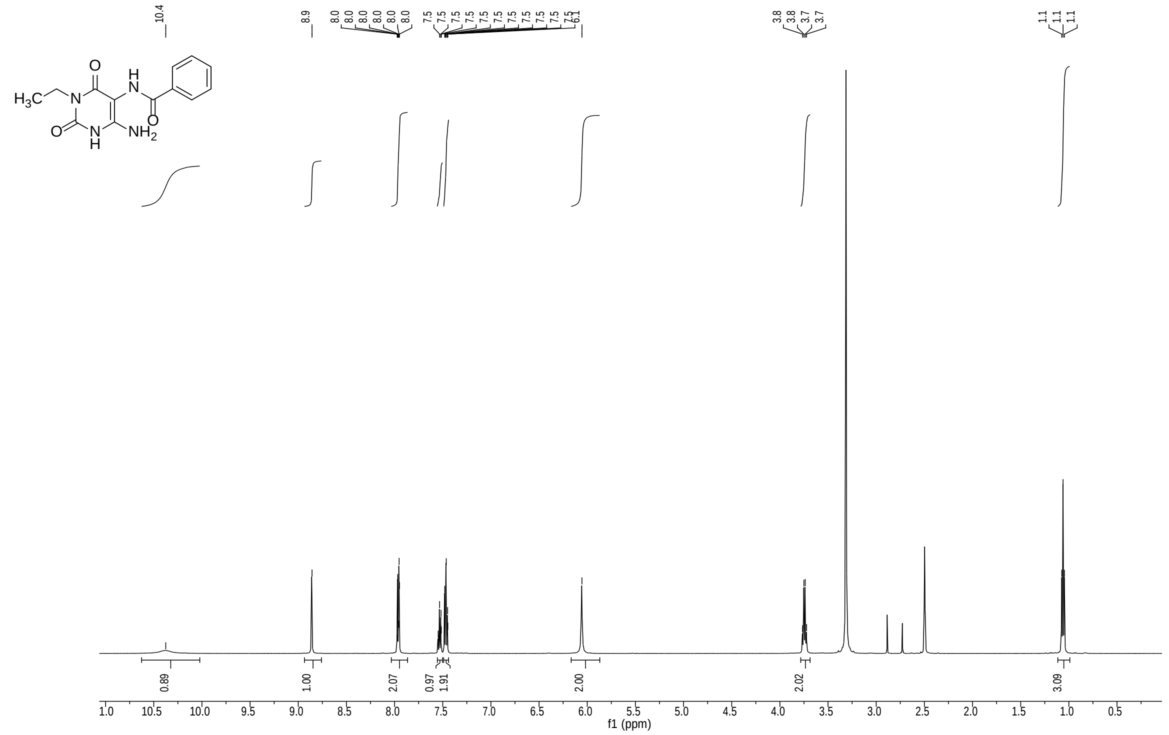

Figure S12. ^{1}H-NMR spectra of **16** in $(CD_3)_2SO$ at room temperature.

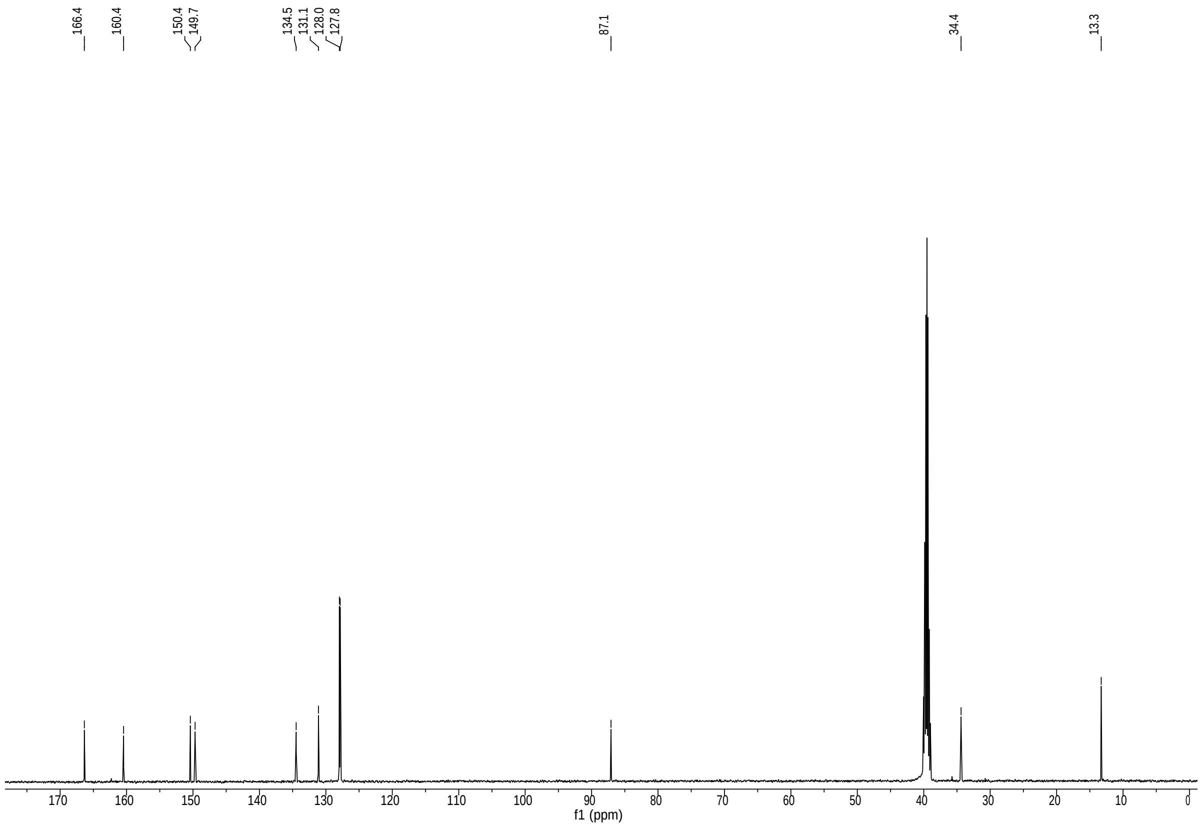

Figure S13. ^{13}C-NMR spectra of **16** in $(CD_3)_2SO$ at room temperature.

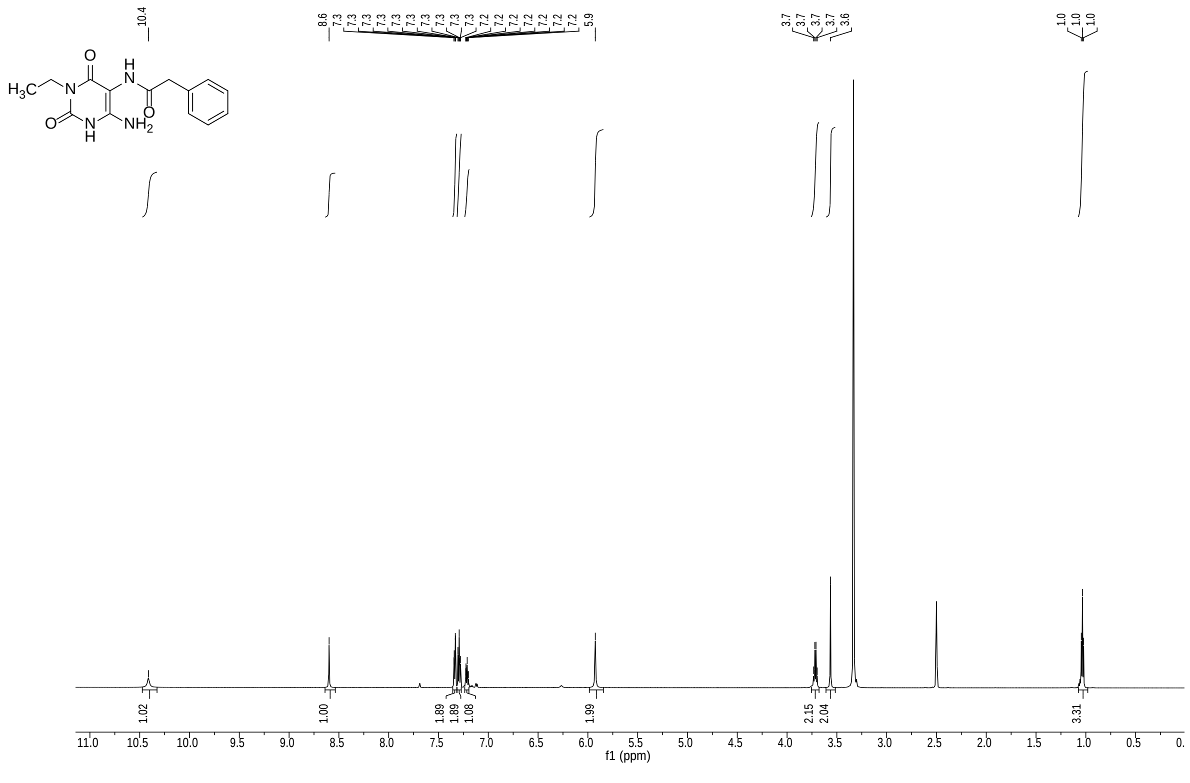

Figure S14. ^{1}H-NMR spectra of **17** in $(CD_3)_2SO$ at room temperature.

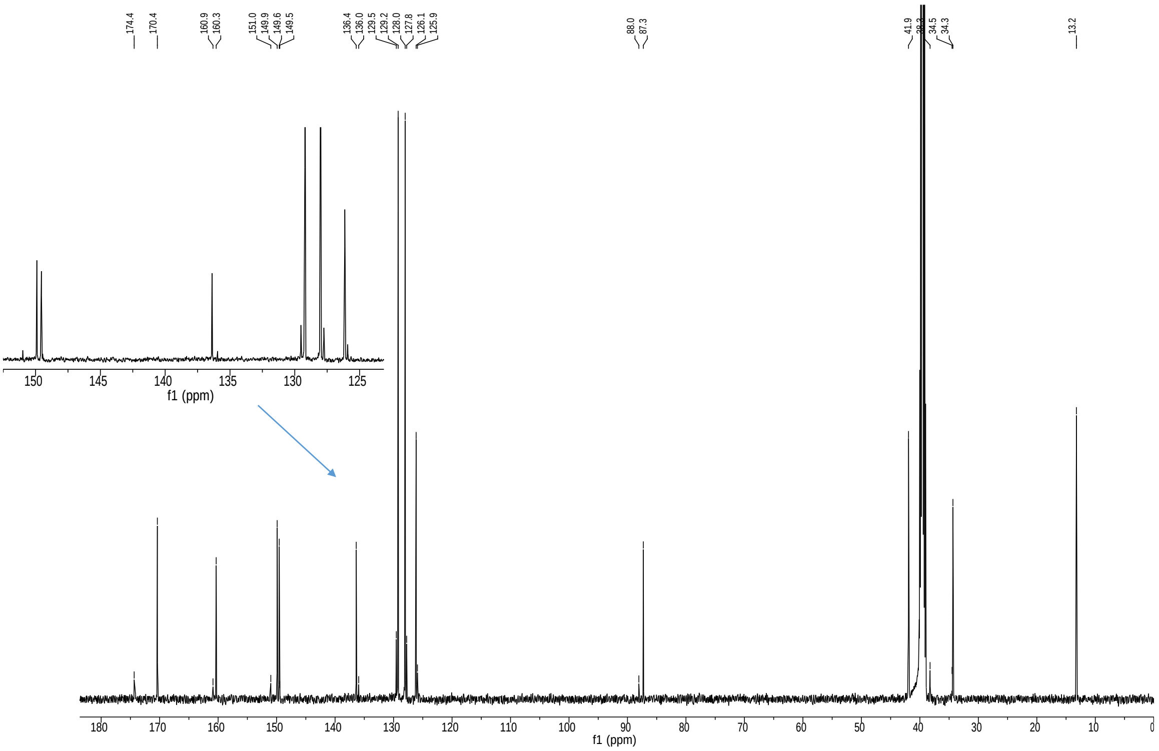

Figure S15. ^{13}C-NMR spectra of **17** in $(CD_3)_2SO$ at room temperature.

DHPLC analyses of **12**

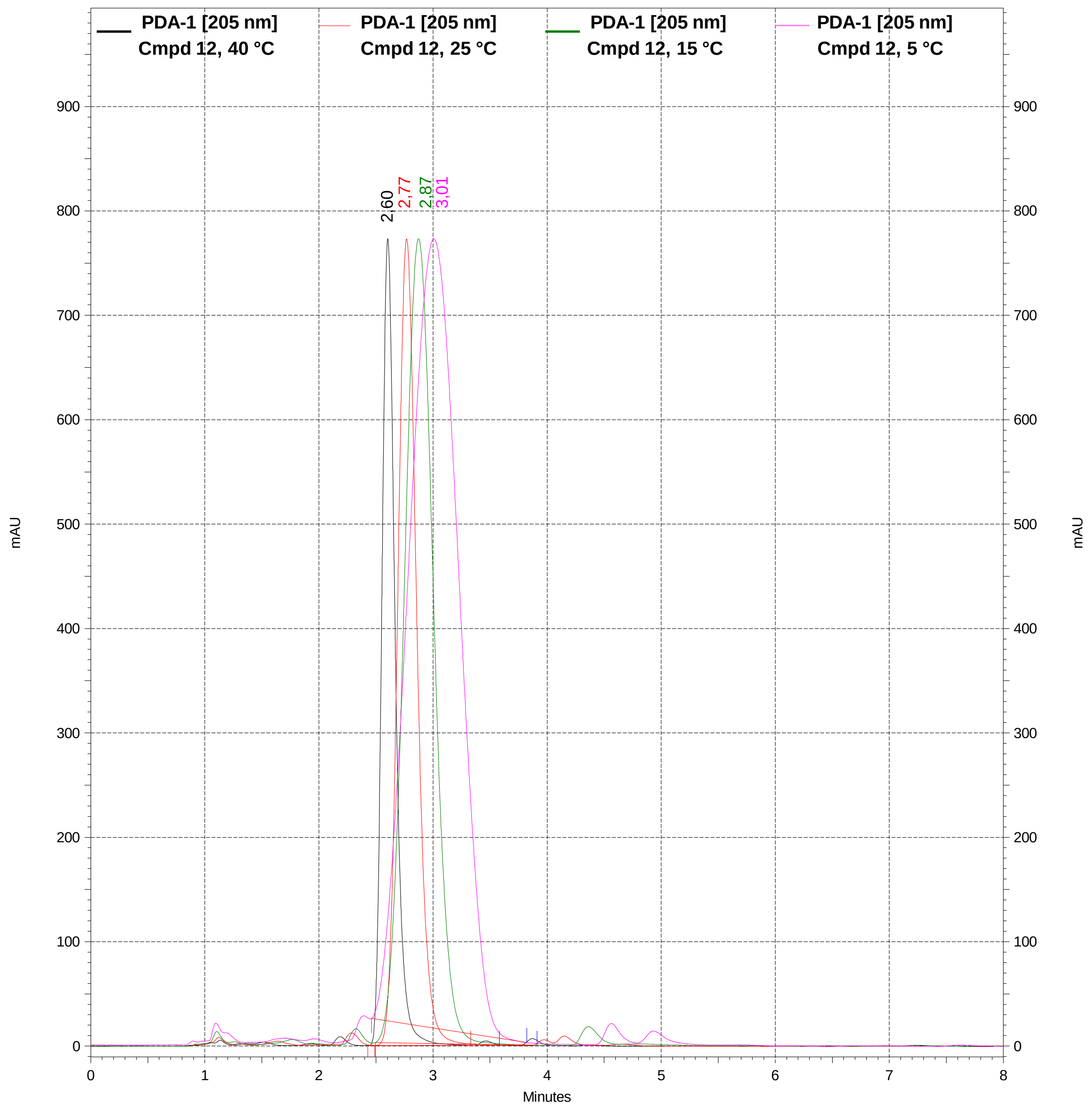

Figure S16: DHPLC analyses of **12** at 205 nm. Compound **12** (2.1 mg) was dissolved in 4.2 ml MeCN and 0.1 µl injected for the DHPLC measurements. A mixture of 40% MeCN and 60% H_2O was used as eluent with a flow rate out of 0.2 ml/min. The pressure increased from 358 bar (25 °C) to 583 bar (5 °C). HPLC chromatogram at 40 °C is shown in black, at 25 °C in red, at 15 °C in green and at 5 °C in pink. The corresponding retention times shifted from 2.60 min at 40 °C to 3.01 min at 5 °C.

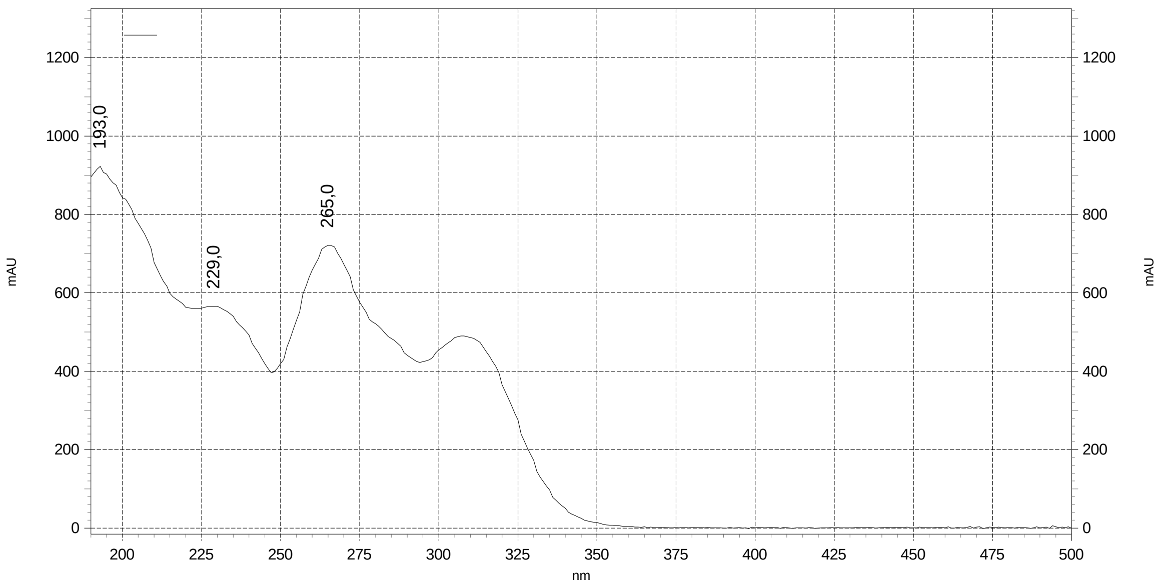

Figure S17: UV-spectra of compound 12

References

1. Hockemeyer, J.; Burbiel, J. C.; Müller, C. E. Multigram-scale syntheses, stability, and photoreactions of A_{2A} adenosine receptor antagonists with 8-styrylxanthine structure: potential drugs for Parkinson's Disease. *J. Org. Chem.* **2004**, 69, 3308-3318.
2. Iii Charles E Maxwell, C. J. S. Method of preparing 4-aminouracils. *US2715625A* **1952**.
3. Marx, D.; Wingen, L. M.; Schnakenburg, G.; Müller, C. E.; Scholz, M. S. Fast, efficient, and versatile synthesis of 6-amino-5-carboxamidouracils as precursors for 8-substituted xanthines. *Frontiers in Chemistry* **2019**, 7, 1-15.
4. Müller, C. E.; Shi, D.; Manning Jr, M.; Daly, J. W. Synthesis of paraxanthine analogs (1, 7-disubstituted xanthines) and other xanthines unsubstituted at the 3-position: structure-activity relationships at adenosine receptors. *J. Med. Chem.* **1993**, 36, 3341-3349.
